# Depletion of the Rho GTPases Cdc42, Rac1 or RhoA reduces PDGF-induced STAT1 and STAT3 signaling

**DOI:** 10.1016/j.bbrep.2024.101828

**Published:** 2024-09-25

**Authors:** Erik Wåhlén, Johan Lennartsson, Johan Heldin

**Affiliations:** Department of Pharmaceutical Biosciences, Uppsala University, Husargatan 3, SE-75124, Uppsala, Sweden

**Keywords:** PDGFR, Cdc42, Rac1, RhoA, Rho GTPases, STAT

## Abstract

This study investigates the role of Rho GTPases, specifically Cdc42, Rac1, and RhoA, in platelet-derived growth factor receptors (PDGFRα and PDGFRβ) signaling. Signal transducer and activator of transcription (STAT) proteins, essential for cellular processes such as proliferation and immune response, are activated downstream of PDGFRs. Dysregulation of these pathways is linked to various diseases, including cancer. The current study examines the effects of Rho GTPase depletion on PDGFR phosphorylation, STAT protein stability, and downstream signaling. Results indicate that depletion of Cdc42, Rac1, or RhoA impairs PDGFR phosphorylation and reduces STAT1 and STAT3 signaling, without significantly affecting AKT and ERK1/2 pathways. The findings highlight the critical regulatory roles of Rho GTPases in PDGFR-mediated STAT signaling.

## Highlights

• STAT1 protein stability is dependent on Cdc42, Rac1 and RhoA.
• Activation of STAT1 and STAT3 was inhibited due to Cdc42, Rac1 and RhoA depletion.
• Cdc42, Rac1 and RhoA depletion decreases PDGFRα and PDGFRβ phosphorylation.

## 1 Introduction

The signal transducer and activator of transcription (STAT) proteins are a family of transcription factors that regulate crucial cellular functions, including cell survival, proliferation, differentiation and immune response. Dysregulation of STAT proteins is frequently found in diseases such as cancer and autoimmune disease, including myeloproliferative neoplasms, lymphomas, and rheumatoid arthritis. The STAT pathway is activated by cytokines and growth factors, including platelet-derived growth factors (PDGFs) [1]. STATs are recruited and phosphorylated, often by Janus kinases (JAKs), leading to STAT dimerization and translocation to the nucleus, where they function as transcription factors [1]. Among the STAT proteins, STAT3 and STAT5 are notably dysregulated in cancers [2]. STAT1 expression is linked to a better prognosis in cancers such as breast cancer and Wilms tumor [1].

Platelet-derived growth factor receptors (PDGFRs) are part of the receptor tyrosine kinase (RTK) family of cell surface receptors [3]. PDGFs are important during embryonal development, being secreted from epithelial and endothelial cells to stimulate PDGFRs on surrounding stromal cells to promote proliferation, migration and differentiation [4]. In adults, the PDGFRs are vital for maintaining tissue homeostasis, including maintaining vascular integrity and interstitial fluid pressure regulation.

PDGFR exists in two isoforms, PDGFRα and PDGFRβ, which have distinct yet overlapping functions [[4], [5], [6]]. Receptor activation occurs through binding with platelet-derived growth factors (PDGFs), which can form AA-, BB-, CC-, -DD homodimers, or an AB-heterodimer [7,8]. These ligands bind to the receptors with different affinities, with PDGF-BB capable of binding and activating both receptor isoforms. Ligand binding induces homo- or heterodimerization of the receptors, resulting in PDGFRαα, PDGFRββ, or PDGFRαβ dimers [7,8]. This dimerization enables transphosphorylation of tyrosine residues on the intracellular domains of the receptors. These phosphorylated residues function as docking sites for SH2-domain-containing proteins, leading to the activation of several downstream signaling pathways, such as the AKT/mTOR, PLCγ, STAT, RAS/RAF/MEK/ERK pathways [4,8].

Rho GTPases are part of the RAS GTPase protein superfamily [9]. These proteins regulate a wide range of cellular functions, including cell migration, polarity, protein trafficking, transformation, survival, and morphogenesis [10]. Approximately half of the known tyrosine kinase receptors have been found to activate different or overlapping sets of Rho GTPases [11]. Among these, RhoA, Rac1, and Cdc42 are most extensively characterized, belonging to the canonical Rho GTPases, which alternate between an active GTP-bound state and inactive GDP-bound state [9]. Cdc42, Rac1, and RhoA play significant roles in cellular trafficking, mainly through their role in regulating the actin cytoskeleton as well as exocytosis and endocytosis [12].

Rho GTPases play a crucial role in regulating the stability of certain receptor tyrosine kinases. For instance, activation of Cdc42 stabilizes the activated EGFR through a mechanism involving GTP-bound Cdc42 and β-PIX, which sequester the E3 ubiquitin ligase c-Cbl, thereby preventing it from ubiquitinating EGFR. This reduction in EGFR ubiquitination leads to a decreased endocytosis and subsequent degradation of the receptor [13]. Similarly, RhoA has been observed to influence EGFR stability, by reducing receptor endocytosis through the action of ROCK, a key regulator in clathrin-mediated endocytosis [10,14]. Additionally, depletion of Cdc42-specific RhoGEF FGD5 increases the ubiquitination of VEGFR2 and enhances its degradation [15].

Given previous reports on the roles of Rho GTPases in the regulation of the stability and activity of various tyrosine kinase receptors, this study explores the role of Rho GTPases in PDGFRα and PDGFRβ signaling.

## 2 Results

### 2.1 PDGFR signaling in response to Cdc42 depletion

Cdc42 is known to regulate the activity of various receptor tyrosine kinase (RTK). To explore its role in PDGFR signaling, we depleted Cdc42 in human foreskin fibroblasts (BJ-hTERT) and monitored the activation and downstream signaling of PDGFR (Fig. 1)

**Fig. 1:**
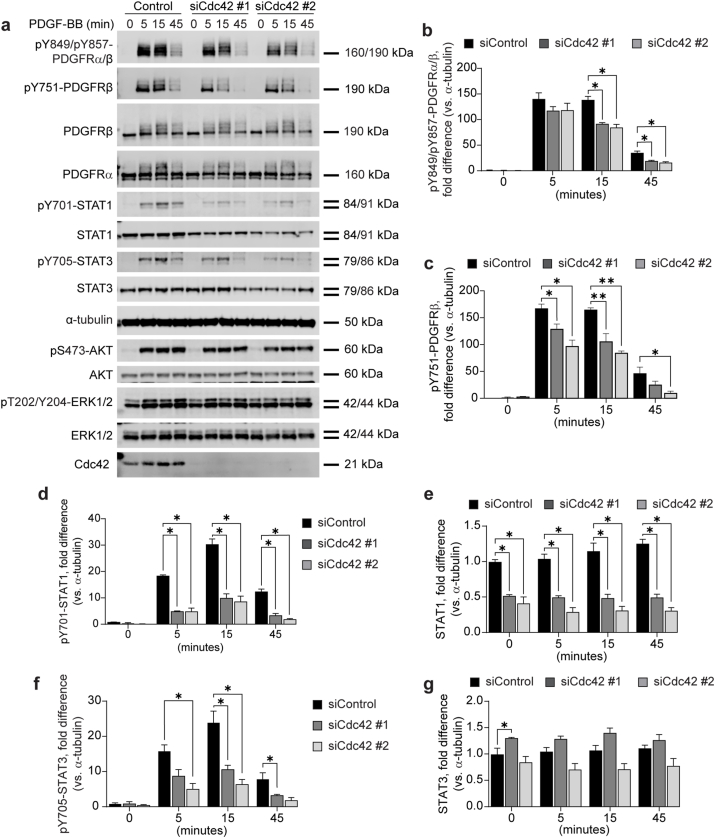
**Knock-down of Cdc42 decreases PDGFR activation and downstream STAT1 and STAT3 activation and STAT1 stability in BJ-hTERT cells.** BJ-hTERT cells were transfected 72 h with siRNAs targeting either Cdc42, or scrambled siRNA, and then stimulated with 20 ng/ml PDGF-BB. Cells were lysed, subjected to SDS-PAGE, and transferred to a PVDF membrane, which was immunoblotted with antibodies targeting proteins shown in (**a**). Activation of PDGFRα/β was monitored and quantified on phosphorylated tyrosine 849 and 857, respectively (**b**), and on phosphorylated tyrosine 751 of PDGFRβ (**c**). Quantification of phosphorylation on tyrosine 701 on STAT1 is shown in (**d**), and total levels of STAT1 in (**e**). Phosphorylation of tyrosine 705 on STAT3 was quantified in (**f**), and total levels of STAT3 in (**g**). Error bars show the standard error of the mean. Quantifications of the immunoblots have been normalized to α-tubulin, and fold difference has been calculated from the mean of all 0 min siControl condition per antibody (n = 4), ∗ = p < 0.05, ∗∗ = p < 0.01.

Using an antibody targeting the phosphorylated tyrosine residue 849 on PDGFRα and tyrosine 857 on PDGFRβ (pY849/pY857-PDGFRα/β), we analyzed PDGFR phosphorylation. Fig. 1 (panels a and b) shows a reduction in phosphorylation of both PDGFR isoforms after 15 and 45 min of stimulation with PDGF-BB. Similarly, phosphorylation of tyrosine residue 751 in the PDGFRβ (pY751-PDGFRβ) was reduced (Fig. 1a–c).

Previous studies indicate that Cdc42 can regulate the stability of EGFR and VEGFR2. We monitored PDGFR stability during different periods of PDGF-BB stimulation with or without Cdc42 depletion (Fig. 1a). The stability of PDGFRα and PDGFRβ did not show a significant difference in Cdc42-depleted cells following PDGF-BB stimulation compared to control (Fig. 1a).

We next assessed the stability and activity of STAT1, STAT3, AKT, and ERK1/2 downstream of activated PDGFRs (Fig. 1a–d-g). Cdc42 depletion did not affect the stability or activity of either ERK1/2 or AKT. However, STAT1 stability and phosphorylation (pY701-STAT1) decreased after Cdc42 depletion (Fig. 1a–d-e). In contrast, STAT3 phosphorylation (pY705-STAT3) was markedly reduced, but here no significant changes in total STAT3 stability were evident compared to controls (Fig. 1a–f-g).

### 2.2 PDGFR signaling in response to Rac1 depletion

**Fig. 2:**
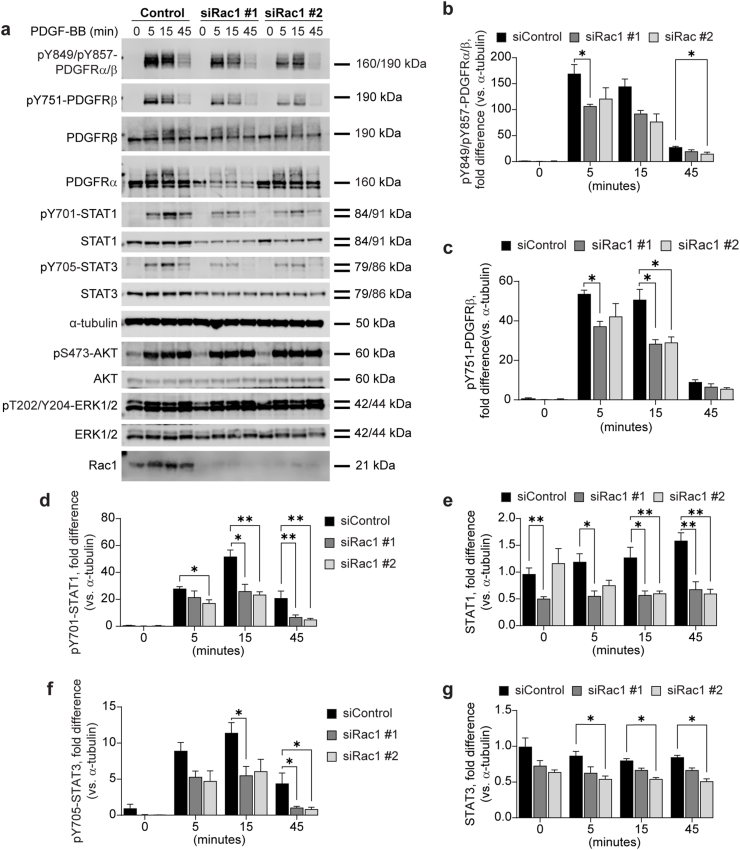
**Knock-down of Rac1 decreases PDGFR activation and downstream STAT1 and STAT3 activation and stability in BJ-hTERT cells.** BJ-hTERT cells were transfected 72 h with siRNAs targeting Rac1, or with scrambled siRNA, and then stimulated with 20 ng/ml PDGF-BB. Cells were lysed, subjected to SDS-PAGE, and transferred to a PVDF membrane, which was immunoblotted with antibodies targeting proteins shown in (**a**). Activation of PDGFRα/β was monitored and quantified on phosphorylated tyrosine 849 and 857, respectively (**b**), and on phosphorylated tyrosine 751 of PDGFRβ (**c**). Quantification of phosphorylation on tyrosine 701 on STAT1 is shown in (**d**), and total levels of STAT1 in (**e**). Phosphorylation of tyrosine 705 on STAT3 was quantified in (**f**), and total levels of STAT3 in (**g**). Error bars show the standard error of the mean. Quantifications of the immunoblots have been normalized to α-tubulin, and fold difference has been calculated from the mean of all 0 min siControl condition per antibody (b-c, n = 4; d-e, n = 5; f-g, n = 4), ∗ = p < 0.05, ∗∗ = p < 0.01.

To explore the role Rac1 in regulating PDGFR stability and activity, we depleted Rac1 in BJ-hTERT cells and subsequently stimulated them with 20 ng/ml PDGF-BB for 0, 5, 15 or 45 min (Fig. 2a).

Similar to the effect observed with Cdc42 depletion, reduced levels of Rac1 resulted in a decrease in PDGFR phosphorylation (Fig. 2a–c). The stability of both PDGFRα and PDGFRβ was investigated in Rac1-depleted cells. The two Rac1 siRNAs produced differing results for PDGFRα stability, preventing any definitive conclusion (Fig. 2a). However, for PDGFRβ stability, no significant difference was observed compared to the control (Fig. 2a).

Reduced Rac1 expression lowered phosphorylation and total levels of STAT1 in PDGF-BB stimulated cells compared to control (Fig. 2a–d, e). Phosphorylated STAT3 was also decreased following Rac1 depletion (Fig. 2a–f). In contrast to the depletion of Cdc42, Rac1 depletion also reduced total STAT3 levels compared to control (Fig. 2a–g). The levels and phosphorylation of AKT and ERK1/2 in response to PDGF-BB stimulation were not affected by Rac1 depletion (Fig. 2a).

### 2.3 PDGFR signaling in response to RhoA depletion

**Fig. 3:**
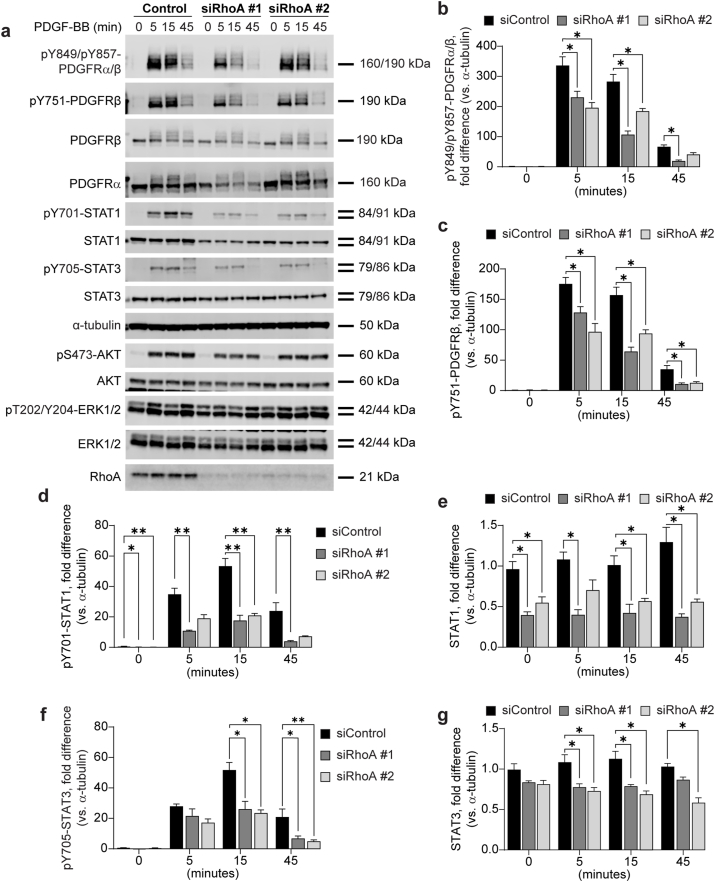
**Knock-down of RhoA decreases PDGFR activation and downstream STAT1 and STAT3 activation and stability in BJ-hTERT cells.** BJ-hTERT cells were transfected 72 h with siRNAs targeting RhoA, or with scrambled siRNA, and stimulated with 20 ng/ml PDGF-BB. Cells were lysed, subjected to SDS-PAGE, and transferred to a PVDF membrane, which was immunoblotted with antibodies targeting proteins shown in (**a**). Activation of PDGFRα/β was monitored and quantified on phosphorylated tyrosine 849 and 857, respectively (**b**), and on phosphorylated tyrosine 751 of PDGFRβ (**c**). Quantification of phosphorylation on tyrosine 701 on STAT1 is shown in (**d**), and total levels of STAT1 in (**e**). Phosphorylation of tyrosine 705 on STAT3 was quantified in (**f**), and total levels of STAT3 in (**g**). Error bars show the standard error of the mean. Quantifications of the immunoblots have been normalized to α-tubulin, and fold difference has been calculated from the mean of all 0 min siControl condition per antibody (b-c, n = 4; d, n = 5; e-g, n = 4), ∗ = p < 0.05, ∗∗ = p < 0.01.

The role of RhoA in PDGFR signaling was investigated by depleting RhoA in BJ-hTERT cells using siRNA. Reduced levels of RhoA led to decreased phosphorylation at all tyrosine residues investigated of both PDGFRα and PDGFRβ in response to PDGF-BB stimulation (Fig. 3a–c).

The effect of RhoA depletion on PDGFRα stability varied depending on the siRNA being used, resulting in inconclusive data (Fig. 3a). No significant difference in the total levels of PDGFRβ was observed between RhoA-depleted cells and controls (Fig. 3a).

In cells with reduced RhoA expression, levels and phosphorylation of STAT1 were diminished compared to controls (Fig. 3a–d, e). Phosphorylation of STAT3 was similarly reduced (Fig. 3a–f). Total STAT3 levels were decreased, albeit less markedly than STAT1 levels (Fig. 3a–g). There were no significant differences in the activation or total levels of AKT and ERK1/2 between RhoA-depleted cells and controls following PDGF-BB stimulation (Fig. 3a).

### 2.4 PDGFR dimerization is not affected in response to Rho GTPase depletion

**Fig. 4:**
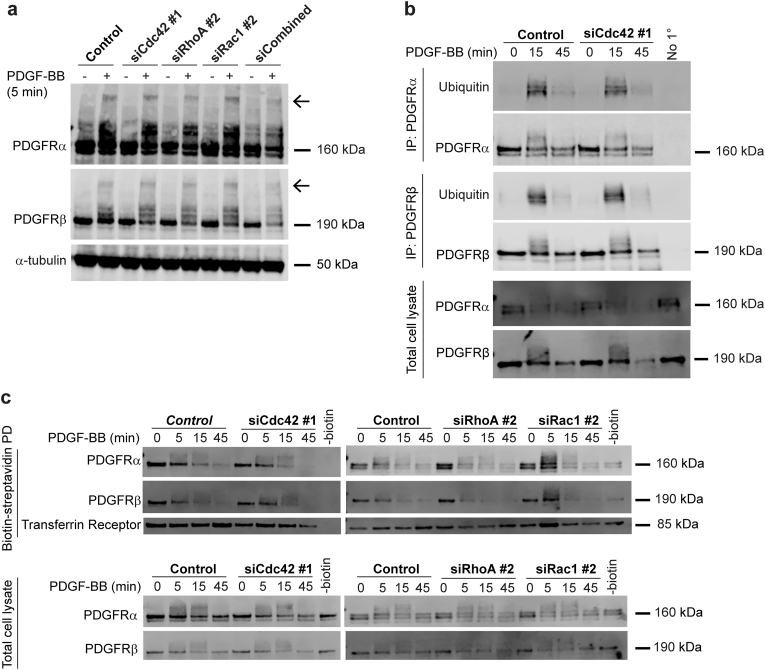
**Knock-down of Cdc42, RhoA or Rac1 does not inhibit PDGFR dimerization, ubiquitination or Internalization.** BJ-hTERT cells were transfected with the indicated siRNAs targeting Cdc42, RhoA or Rac1, or with scrambled siRNA, and after 72 h cells were stimulated with 20 ng/ml PDGF-BB. (**a**) PDGFR dimers were cross-linked with BS3 and subjected to SDS-PAGE, after which proteins were immunoblotted for specific antibodies against PDGFRα, PDGFRβ and α-tubulin (receptor dimer is indicated with an arrow). (**b**) PDGFRα or -β was immunoprecipitated using isoform-specific antibodies targeting the receptors. Lysates were then subjected to SDS-PAGE and then immunoblotted with specific antibodies against PDGFRα, PDGFRβ and ubiquitin. No 1° = No primary antibody added. (PDGFRα, n = 4; PDGFRβ, n = 3). (**c**) Cell surface biotinylated proteins were precipitated and subjected to SDS-PAGE and then immunoblotted with antibodies against PDGFRα, PDGFRβ and Transferrin Receptor (TfR). -Biotin = No cell surface biotinylation. (n = 4).

To determine whether the expression of Cdc42, Rac1 or RhoA influences PDGFR dimerization, we depleted the Rho GTPases individually and in combination in BJ-hTERT fibroblasts. Following depletion, cells were stimulated with PDGF-BB, and PDGFR dimerization was assessed using BS3 cross-linking and receptor dimers was detected by the increased molecular weight of the PDGFRs (Fig. 4a). Under these experimental conditions, no significant effect on PDGFRα or PDGFRβ dimerization could be observed (Fig. 4a).

### 2.5 PDGFR ubiquitination is not affected in response to Rho GTPase depletion

To investigate if PDGFR ubiquitination is regulated by Cdc42, as previously suggested for EGFR and VEGFR2 [13,15], we conducted experiments in BJ-hTERT cells depleted of Cdc42. After depletion, PDGFRα or PDGFRβ were immunoprecipitated using isoform-specific antibodies. The ubiquitination levels of the receptor were monitored by immunoblotting using a ubiquitin antibody. Our findings suggest that the ubiquitination levels of neither PDGFRα nor PDGFRβ were substantially affected by the depletion of Cdc42 (Fig. 4b).

### 2.6 PDGFR internalization is inhibited in response to Rho GTPase depletion

To determine whether the levels of PDGFR at the plasma membrane are regulated by Cdc42, Rac1, or RhoA, we depleted these Rho GTPases in BJ-hTERT cells and analyzed PDGFR internalization.

Cell surface proteins were labeled with biotin after various periods of PDGF-BB stimulation and precipitated using streptavidin-coated beads. This allowed us to assess the levels of PDGFRα and PDGFRβ at the plasma membrane. Our analysis revealed that depletion of RhoA, but not Cdc42 and Rac1, increased levels of both PDGFRα and PDGFRβ at the plasma membrane during unstimulated conditions (Fig. 4c).

Following stimulation with PDGF-BB we did not observe significant differences in the internalization of either PDGFRα or PDGFRβ from the plasma membrane in cells with reduced levels of Cdc42, RhoA or Rac1 compared to control (Fig. 4c).

## 3 Discussion

In this study, we investigated the effects of depletion of the Rho GTPases Cdc42, Rac1 and RhoA on phosphorylation and downstream signaling of PDGFRα and PDGFRβ.

Our observations indicate that the depletion of Cdc42, Rac1 or RhoA negatively impacts the activation of PDGFRβ, as evidenced by reduced pY751-PDGFRβ levels (Fig. 1, Fig. 2, Fig. 3). Although we lack an antibody specific for monitoring PDGFRα phosphorylation, the reduced signal from the pY849/pY857-PDGFRα/β antibody, which recognizes the phosphorylated activation loop in both PDGFRα and PDGFRβ, suggests that PDGFRα phosphorylation is also likely reduced when Cdc42, RhoA or Rac1 protein levels are depleted (Fig. 1, Fig. 2, Fig. 3).

Previous studies have shown that the depletion of Rho GTPases regulates the protein stability of other RTKs, such as EGFR and VEGFR2, by modulation of activity of E3 ubiquitin ligases [13,15]. It has been proposed that activated Cdc42, together with β-PIX, can sequester the E3-ubiquitin ligase c-Cbl, thus preventing EGFR ubiquitination and subsequent degradation [13]. Similarly, FGD5, which activates Cdc42, has been suggested to protect VEGFR2 from ubiquitination and degradation [15]. However, our findings were unable to conclusively confirm that Cdc42 has the same effect on PDGFRs stability or ubiquitination as reported for EGFR and VEGFR2 (Fig. 1, Fig. 4). Additionally, we could not definitively determine that RhoA or Rac1 exert a similar protective effect on the PDGFRs (Fig. 2, Fig. 3).

Given the reduced activity of PDGFRα and PDGFRβ following the depletion of Cdc42, Rac1 or RhoA, we investigated whether Rho GTPases play a role in regulating receptor dimerization of PDGFRα and PDGFRβ, as previously shown for PDGFRβ in response to inhibition of large GTPase Dynamin II [16]. Using BS3 cross-linking to monitor receptor dimerization, we found that depletion of Cdc42, Rac1 or RhoA did not significantly affect PDGFRα or PDGFRβ (Fig. 4).

Since Rho GTPases are also known to regulate different endocytosis pathways we wanted to investigate their role in PDGFR degradation. We observed increased steady-state levels of both PDGFRα and PDGFRβ, upon the depletion of RhoA, but not Cdc42 and Rac1 (Fig. 4). This suggests that RhoA plays a role in maintaining these receptors at the plasma membrane, contrasting a previous study using RhoA, RhoB and RhoC inhibitors where no difference in PDGFRβ levels was detected using an immunofluorescence readout [17].

We also observed that activation of STAT1 and STAT3 by PDGFRα and PDGFRβ was negatively impacted by silencing of Cdc42, Rac1, or RhoA (Fig. 1, Fig. 2, Fig. 3). For STAT1, this likely results from decreased protein stability seen following Cdc42, Rac1, and RhoA depletion, reducing the available protein pool for activation. However, total STAT3 levels were not similarly affected by Cdc42 depletion, though they were markedly reduced by Rac1 and especially RhoA depletion. A previous study similarly indicated that PDGFRβ-mediated STAT3 activation is inhibited by Cdc42 depletion [17]. The same study also showed that Cdc42/Rac1 inhibitor ML141 reduced STAT3 phosphorylation, which is in agreement with our findings that both Cdc42 and Rac1 depletion substantially reduce of STAT3 phosphorylation. Furthermore, inhibition of ROCK1/2 activity downstream RhoA, was shown to decrease STAT3 phosphorylation, consistent with our observations. That study attributed this to the reduced internalization of PDGFRβ [17]. Our data suggests that this reduction is attributed, at least in part, to the reduced activation of PDGFRα and PDGFRβ (Fig. 1, Fig. 2, Fig. 3) and/or decreased STAT3 protein levels seen after RhoA and Rac1 depletion. The mechanism behind the reduced STAT1 and STAT3 protein levels is unclear, but may involve Rho GTPases regulating STAT stability similarly to their reported role in EGFR stability.

While Cdc42, Rac1, and RhoA depletion negatively affected STAT1 and STAT3 phosphorylation downstream of PDGFRα and PDGFRβ, it did not significantly impact AKT and ERK1/2 signaling (Fig. 1, Fig. 2, Fig. 3). This may relate to the absence of intermediary kinase signal amplification in STAT activation, unlike ERK1/2 and AKT pathways, which involve multiple upstream protein kinases [18,19].

Several STAT family members interact with different Rho GTPases. Cdc42, Rac1 and RhoA has been shown to increase expression and phosphorylation of STAT3 and STAT5 [2]. Additionally, RhoH influences STAT activation in hematopoietic cells, where high RhoH expression is associated with increased STAT1 activation, while low RhoH expression induces STAT5A activation [2].

In summary, our study underscores the roles of Cdc42, Rac1, and RhoA in regulating PDGFRα and PDGFRβ signaling. We observed that these Rho GTPases significantly impair PDGFR activation and downstream STAT1 and STAT3 phosphorylation while not impacting ERK1/2 and AKT pathways.

## 4 Materials & methods

### 4.1 Cell culture

BJ-hTERT fibroblasts were cultured with DMEM + GlutaMAX with 10 % FBS as described in Ref. [20].

### 4.2 Transfection and siRNA gene silencing

BJ-hTERT cells were transfected with Lipofectamine 3000, according to the manufacturer's instructions (Thermo Fisher Scientific), as described in Ref. [16]. siRNA used in this study was, scrambled negative control (Silencer™ Select Negative Control No. 1 siRNA, 4390843, Thermo Fisher Scientific), siRNA anti-Cdc42 (Silencer™ Select, s2765 (siCdc42 #1), s2767 (siCdc42 #2), Thermo Fisher Scientific), siRNA anti-RhoA (Silencer™ Select, s758 (siRhoA #1), s760 (siRhoA #2), Thermo Fisher Scientific), siRNA anti-Rac1 (Silencer™ Select, s11711 (siRac1 #1), s11712 (siRac1 #2)).

### 4.3 Western blot analysis

Western blot analysis was performed as described in Ref. [21]. In essence, BJ-hTERT cells were seeded to a confluence of 50,000 cells/cm^2^, and transfection of cells with siRNA was performed as described above. After treatments, cells were lysed, denatured at 95 °C for 5 min, and subjected to gel electrophoresis and transferred to a PVDF-FL membrane (Merck). Membranes were then incubated overnight at 4 °C with primary antibodies (described in Supplementary Table 1). Incubated with the appropriate secondary antibodies (Alexa680 or IRDye800) and lastly membranes were scanned with an Odyssey Scanner and quantified with ImageStudio Software v5.2.5 (LI-COR Biosciences).

### 4.4 Cell surface biotinylation assay

Cell surface biotinylation was performed as described earlier [16]. Briefly, BJ-hTERT cells were seeded and transfected with siRNA as described above. After treatments, the cells were incubated in 0.3 mg/ml Sulfo–NHS–Biotin for 120 min at 4 °C and then quenched with TBS. Cells were lysed with modified RIPA buffer (IP lysis buffer) for 15 min on ice. Lysates were cleared and equal volumes of the lysates were incubated together with 50 μl of magnetic streptavidin beads for 60 min at 4 °C. The streptavidin-bound biotinylated proteins were eluted with LDS lysis buffer. Samples were then analyzed as described in the *Western blot analysis* section.

### 4.5 Dimerization assay

Dimerization was performed as described in Ref. [21]. In essence, cells were seeded and transfected with siRNA as described above. After cell treatments, the cells were incubated with 2 mM BS3 in PBS for 60 min on ice. The reaction was then quenched with TBS. Subsequently, cells were lysed in LDS lysis buffer and analyzed as described in the *Western blot analysis* section.

### 4.6 Immunoprecipitation and ubiquitination

BJ-hTERT cells were seeded and siRNA treatments were conducted as described above. After starvation, cells were treated with 20 ng/ml PDGF-BB for either 0, 15 or 45 min at 37 °C. Cells were then placed on ice and washed with ice-cold PBS and thereafter lysed with IP lysis buffer for 15 min on ice. Lysates were cleared by centrifugation at 13,000 rpm and 4 °C and an equal volume of supernatants was saved. 2 μg/ml Goat anti-PDGFR-α IgG or 2 μg/ml Goat anti-PDGFR-β IgG was added to the supernatants and incubated for 120 min at 4 °C. The samples were next incubated with 50 μl protein G magnetic beads (prot. G Mag Sepharose™, GE28-9670-66, GE Healthcare) for 120 min at 4 °C. The supernatants were removed and the magnetic beads were washed with IP lysis buffer, and then bound proteins were eluted with LDS lysis buffer. Samples were then analyzed as described in the *Western blot analysis* section.

### 4.7 Statistical analyzes

Experiments were independently repeated at least four times. For statistical analysis, GraphPad Prism 9 was used. To determine whether data was normally distributed, the Shapiro-Wilks test was employed. Non-parametric statistical analysis (Kurskal-Wallis with a Dunn's post hoc test or Mann-Whitney test) was employed for all analyses.

## Funding

This study has been supported by the 10.13039/501100002794Swedish Cancer Society (J.L., 21 1427 Pj 01H).

## CRediT authorship contribution statement

**Erik Wåhlén:** Formal analysis, Investigation, Validation, Visualization, Writing – original draft, Writing – review & editing. **Johan Lennartsson:** Conceptualization, Funding acquisition, Resources, Supervision, Writing – original draft, Writing – review & editing. **Johan Heldin:** Conceptualization, Project administration, Supervision, Writing – original draft, Writing – review & editing.

## Declaration of competing interest

The authors report there are no competing interests to declare.

## Figures and Tables

**figs1:**
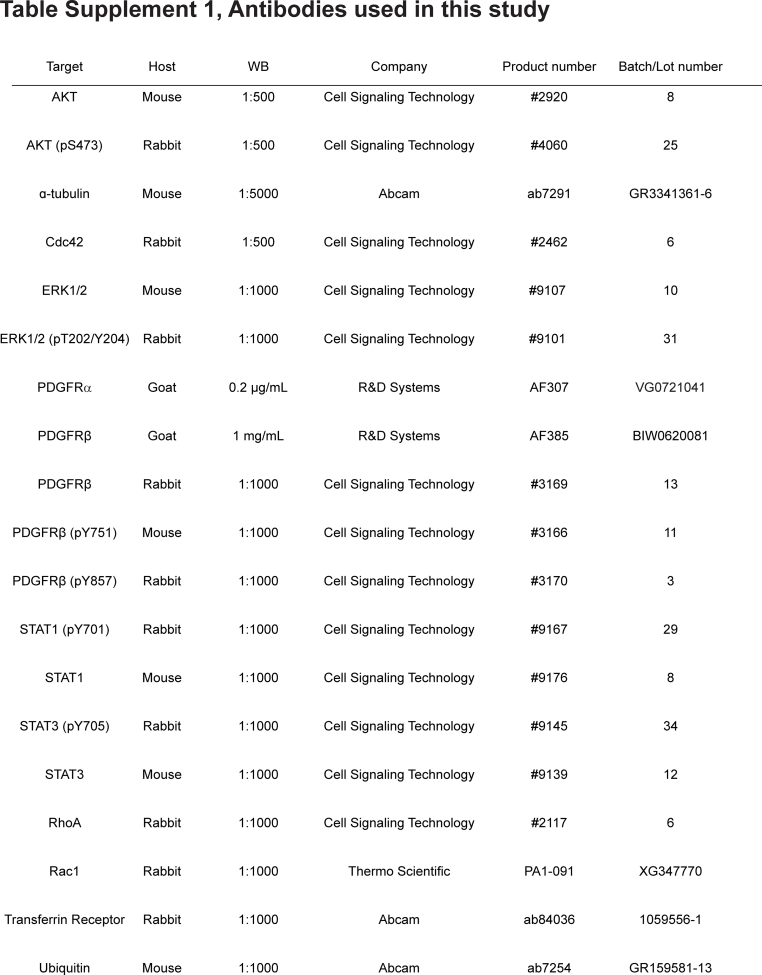


## Data Availability

Data will be made available on request.
