# Supplementary material for: Depletion of the Rho GTPases Cdc42, Rac1 or RhoA reduces PDGF-induced STAT1 and STAT3 signaling
Source: Biochem Biophys Rep. 2024 Sep 25;40:101828. doi: 10.1016/j.bbrep.2024.101828 (PMC11460520; doi:10.1016/j.bbrep.2024.101828)

Rac1 knock-down, experiment 3

Experiment 3, Membrane 1, channel 1

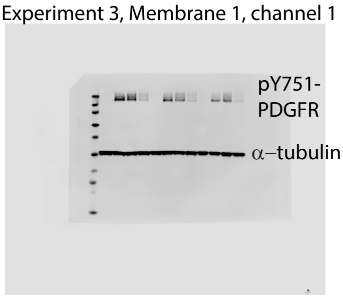

Experiment 3, Membrane 4 , channel 2

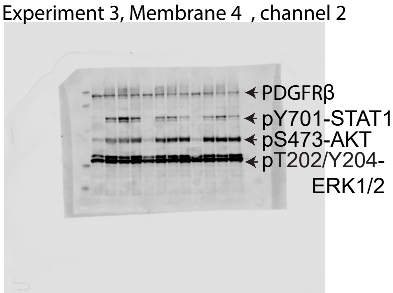

Experiment 3, Membrane 2, channel 2

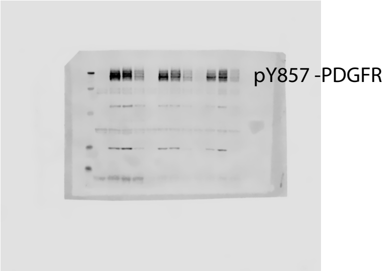

Experiment 3, Membrane 6 , channel 1

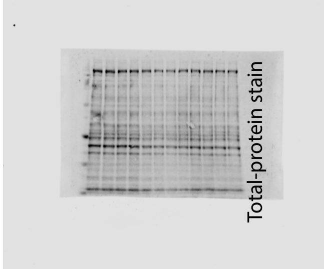

Experiment 3, Membrane 2, channel 2

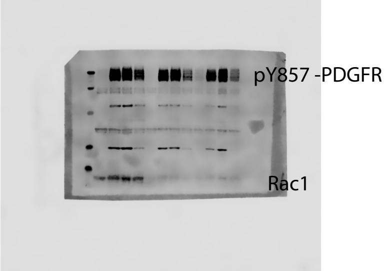

Experiment 3, Membrane 3, channel 1

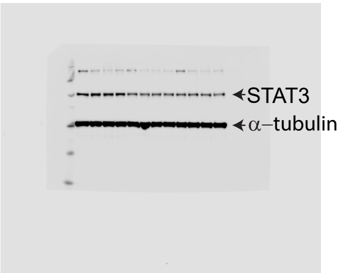

Experiment 3, Membrane 3, channel 2

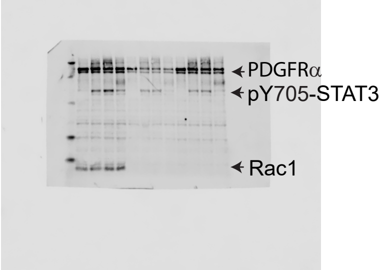

Experiment 3, Membrane 4, channel 1

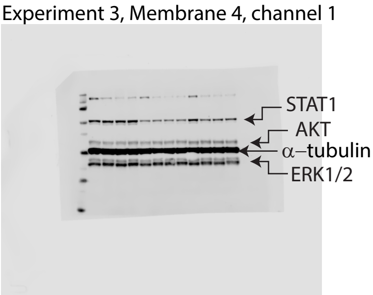

Rac1 knock-down, experiment 4

Experiment 4, Membrane 1, channel 1

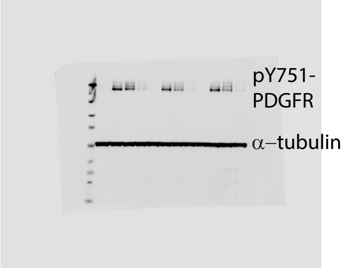

Experiment , Membrane 4, channel 2 (high contrast)

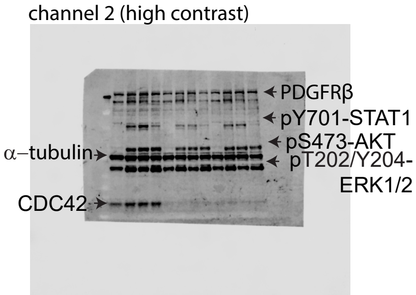

Experiment 4, Membrane 2, channel 2

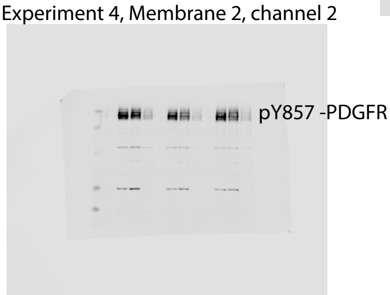

Experiment 4, Membrane 3, channel 1

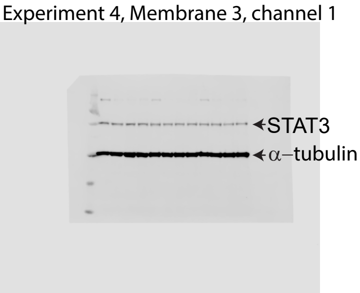

Experiment 4, Membrane 3, channel 2

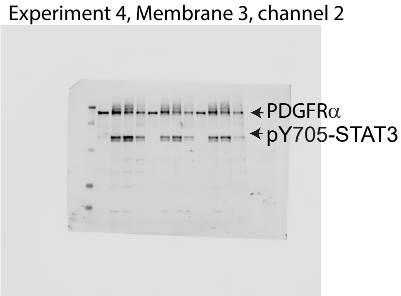

Experiment 4, Membrane 4, channel 1

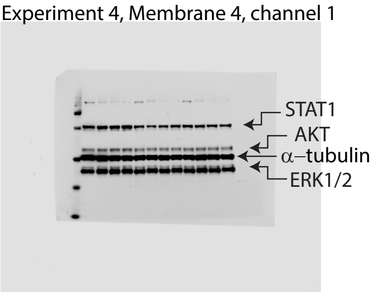

Experiment , Membrane 4 , channel 2

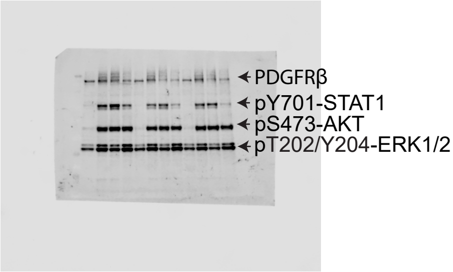

Figure 2, full blots

Rac1 knock-down, experiment 1

Experiment 1, Membrane 1, channel 1

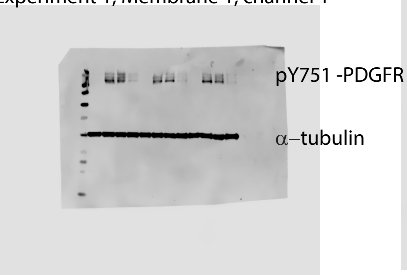

Experiment 1, Membrane 4 , channel 2

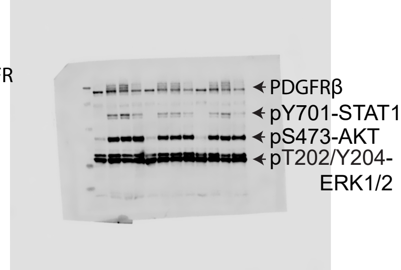

Experiment 1, Membrane 2, channel 1

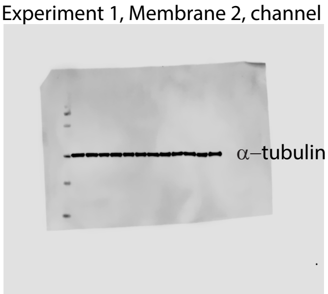

Experiment 1, Membrane 5, channel 1

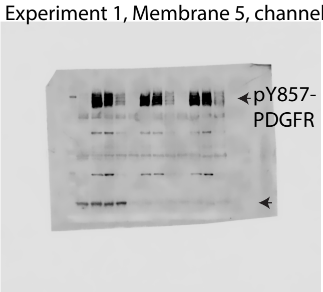

Experiment 1, Membrane 2, channel 2

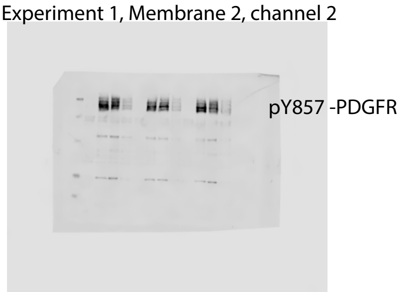

Experiment 1, Membrane 6 , channel 1

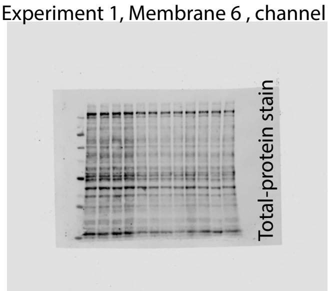

Experiment 1, Membrane 3, channel 1

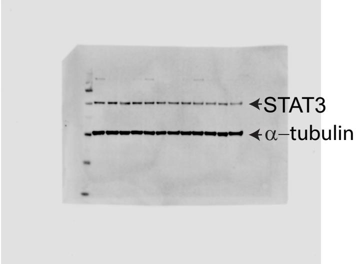

Experiment 1, Membrane 3, channel 2

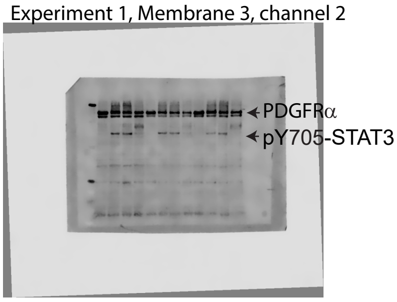

Experiment 1, Membrane 4, channel 1

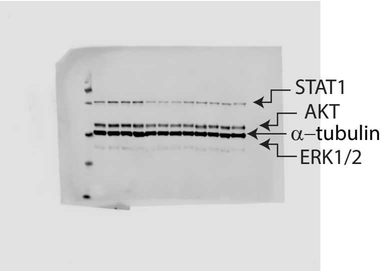

Rac1 knock-down, experiment 2

Experiment 2, Membrane 1, channel 1

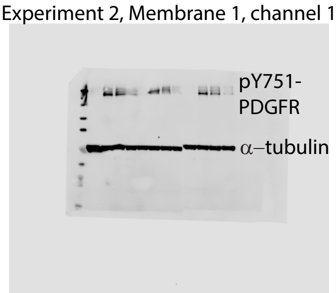

Experiment 2, Membrane 4 , channel 2

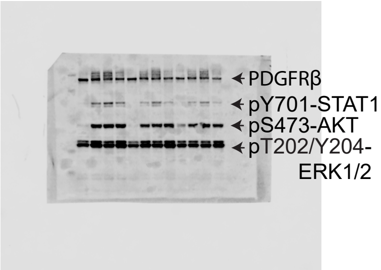

Experiment 2, Membrane 2, channel 1

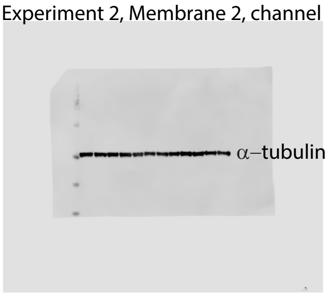

Experiment 2, Membrane 4, channel 2

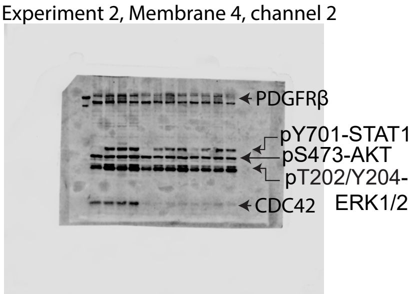

Experiment 2, Membrane 2, channel 2

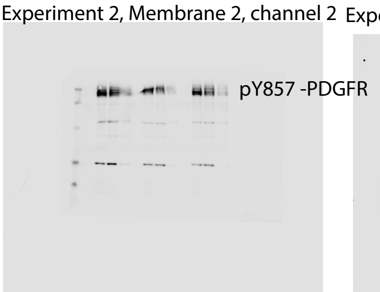

Experiment 2, Membrane 6 , channel 1

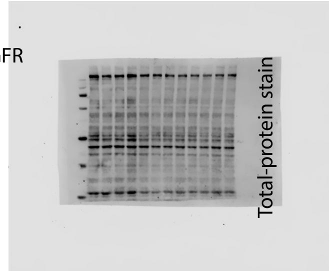

Experiment 2, Membrane 3, channel 1

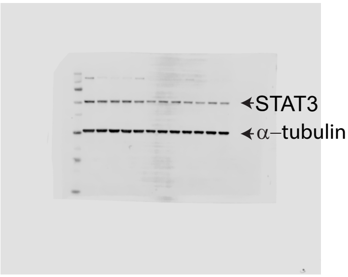

Experiment 2, Membrane 3, channel 2

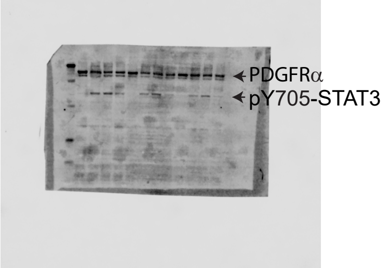

Experiment 2, Membrane 4, channel 1

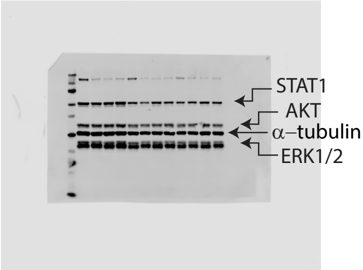

Figure 3, full blots

RhoA knock-down, experiment 1

Experiment 1, Membrane 1, channel 1

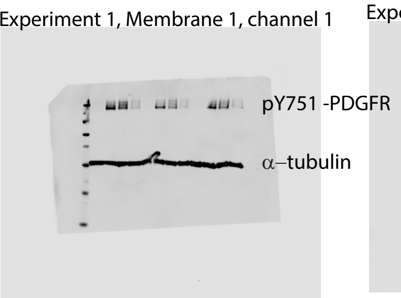

Experiment 1, Membrane 4 , channel 2

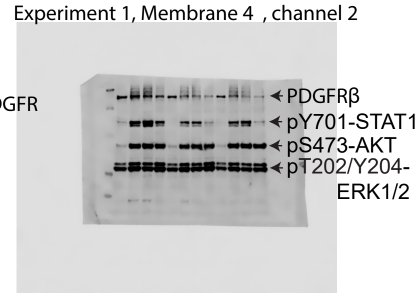

Experiment 1, Membrane 2, channel 1

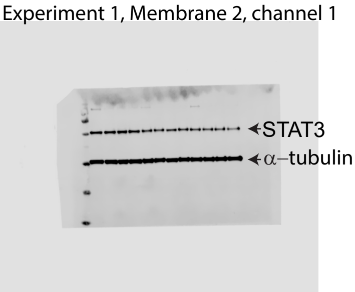

Experiment 1, Membrane 5, channel 1

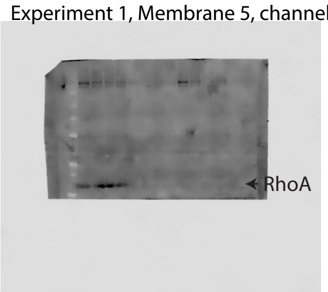

Experiment 1, Membrane 2, channel 2

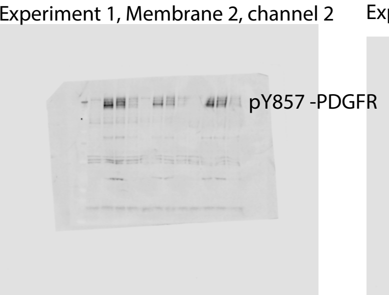

Experiment 1, Membrane 6 , channel 1

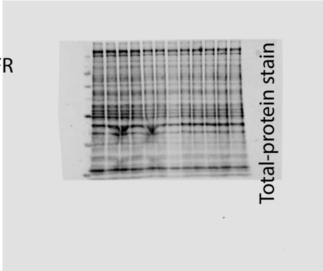

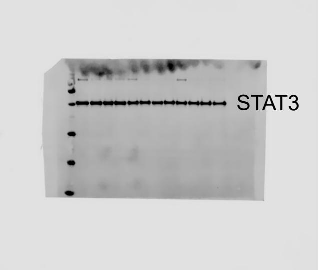

Experiment 1, Membrane 3, channel 2

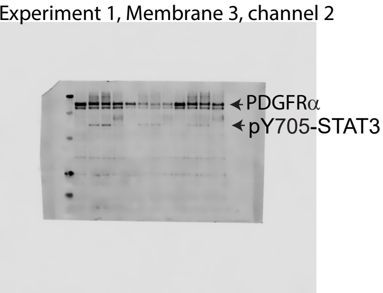

Experiment 1, Membrane 4, channel 1

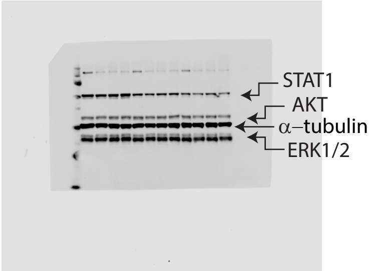

RhoA knock-down, experiment 2

Experiment 2, Membrane 1, channel 1

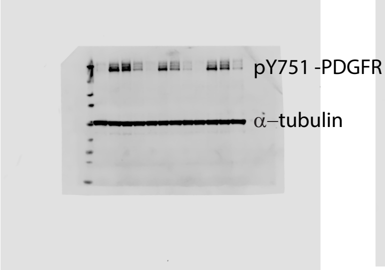

Experiment 2 , Membrane 4 , channel 2

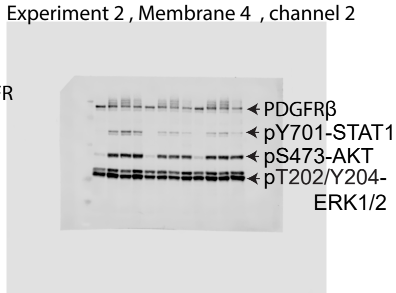

Experiment 2, Membrane 2, channel 1

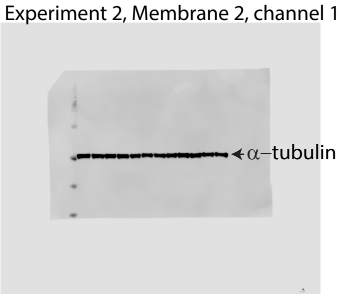

Experiment 2, Membrane 5, channel 1

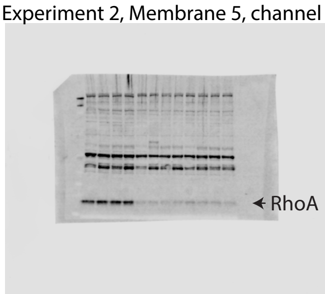

Experiment 2, Membrane 2, channel 2

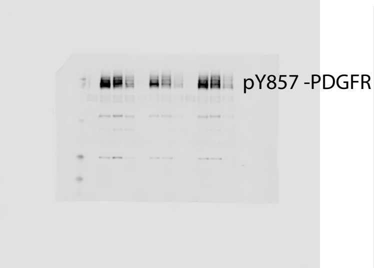

Experiment 2, Membrane 6 , channel 1

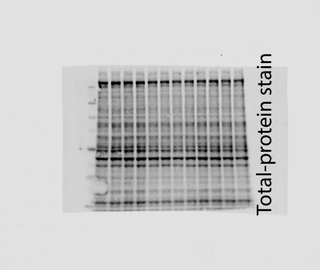

Experiment 2, Membrane 3, channel 1

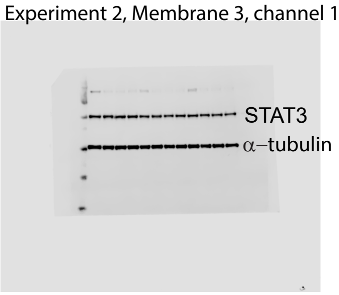

Experiment 2, Membrane 3, channel 2

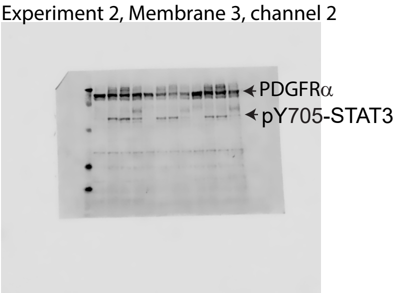

Experiment 2, Membrane 4, channel 1

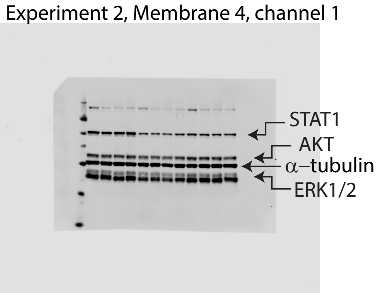

RhoA knock-down, experiment 3

Experiment 3, Membrane 1, channel 1

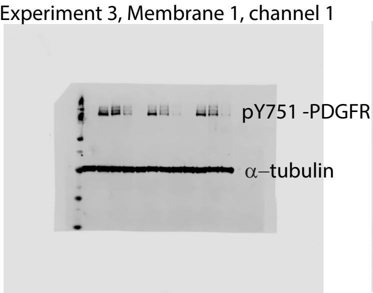

Experiment 3, Membrane 5 , channel 2

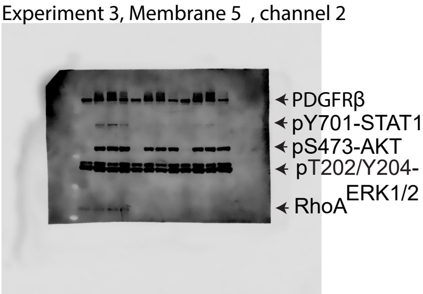

Experiment 3, Membrane 2, channel 2

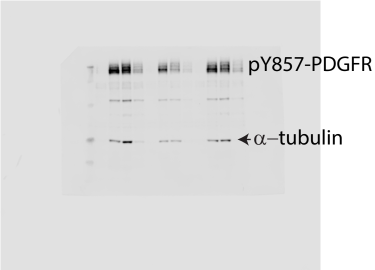

Experiment 3, Membrane 3, channel 1

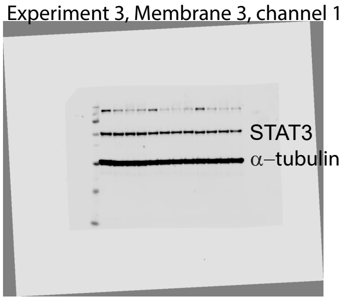

Experiment 3, Membrane 3, channel 2

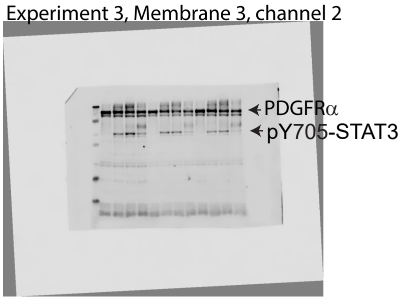

Experiment 3, Membrane 4, channel 1

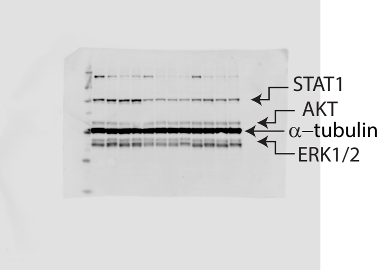

Experiment 3, Membrane 4 , channel 2

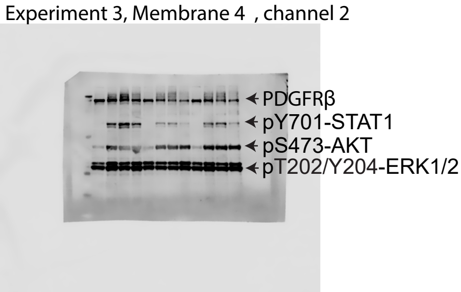

RhoA knock-down, experiment 4

Experiment 4, Membrane 1, channel 1

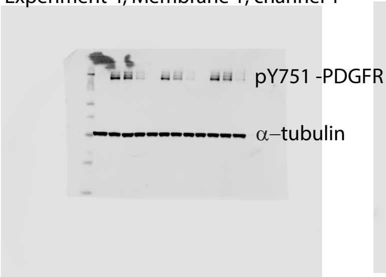

Experiment 4, Membrane 5 , channel 2

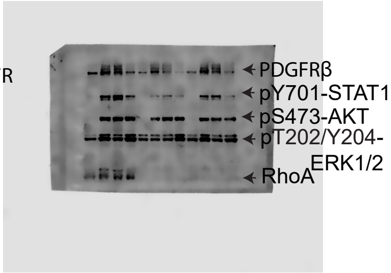

Experiment 4, Membrane 2, channel 2

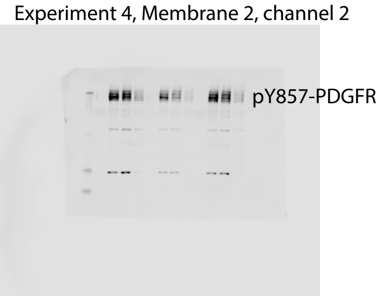

Experiment 4, Membrane 3, channel 1

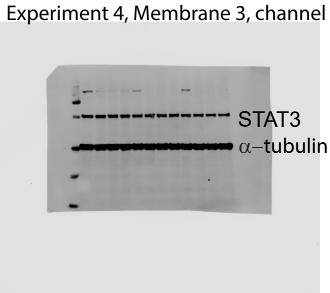

Experiment 4, Membrane 3, channel 2

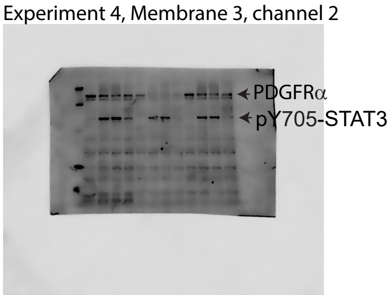

Experiment 4, Membrane 4, channel 1

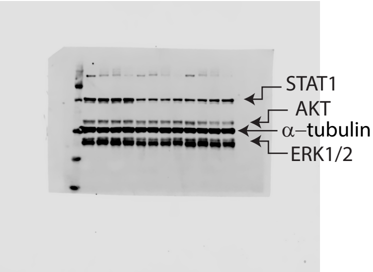

Experiment 4, Membrane 4 , channel 2

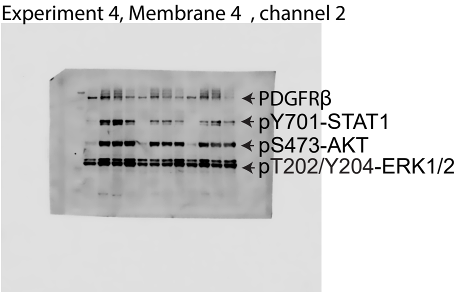

Figure 1, full blots

CDC42 knock-down, experiment 1

Experiment 1, Membrane 1, channel 1

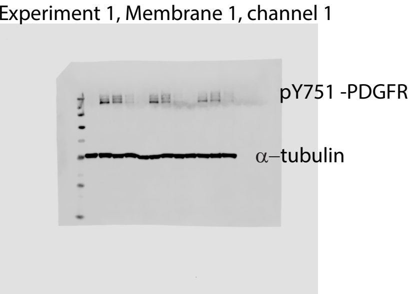

Experiment 1, Membrane 2, channel 1

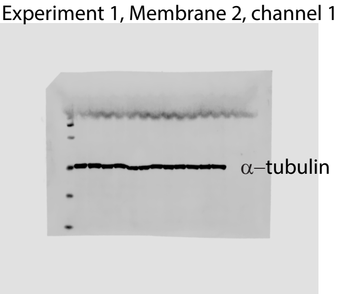

Experiment 1, Membrane 2, channel 2

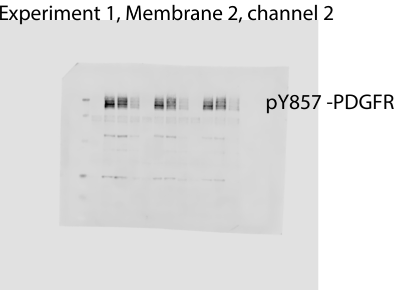

Experiment 1, Membrane 3, channel 1

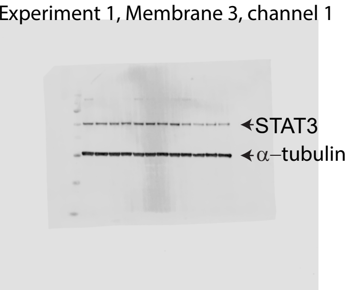

Experiment 1, Membrane 3, channel 2

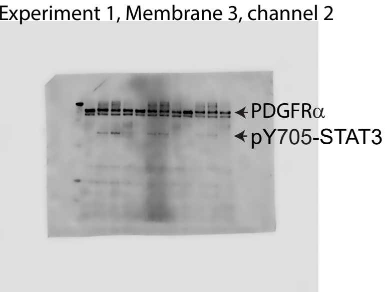

Experiment 1, Membrane 4, channel 1

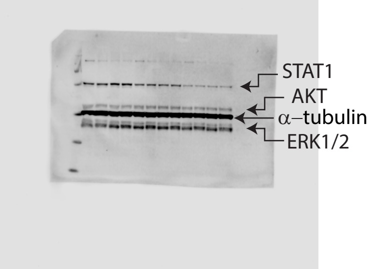

Experiment 1, Membrane 4 , channel 2

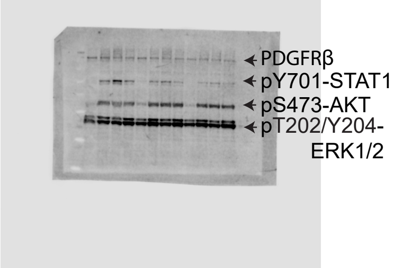

Experiment 1, Membrane 5, channel 1

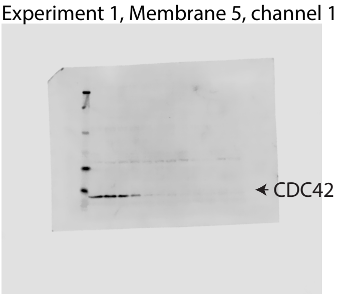

Experiment 1, Membrane 6 , channel 1

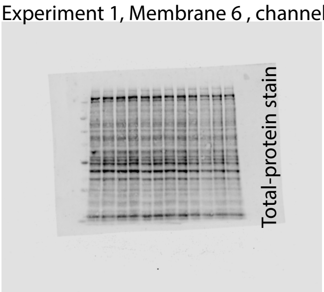

CDC42 knock-down, eperiment 2

Experiment 2, Membrane 1, channel 1

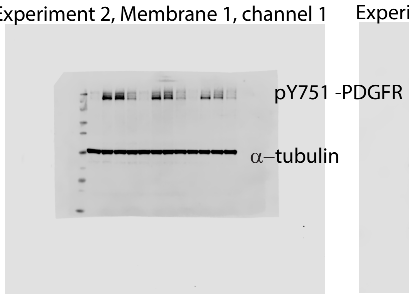

Experiment 2, Membrane 2, channel 1

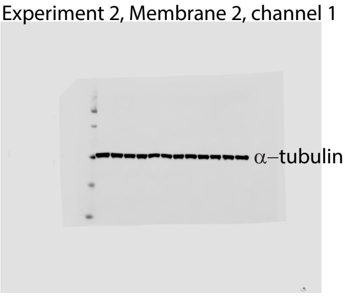

Experiment 2, Membrane 2, channel 2

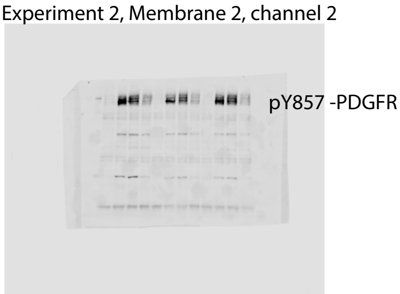

Experiment 2, Membrane 3, channel 1

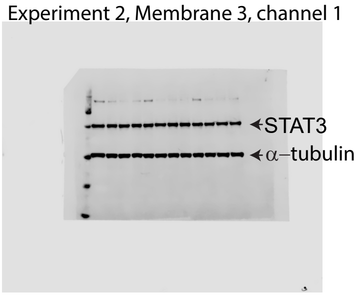

Experiment 2, Membrane 3, channel 2

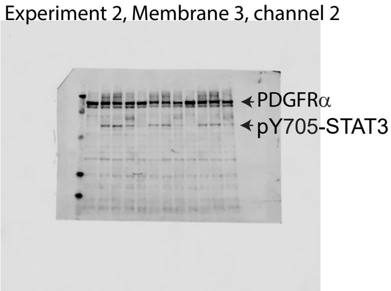

Experiment 2, Membrane 4, channel 1

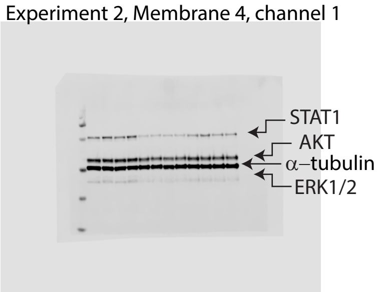

Experiment 2, Membrane 4 , channel 2

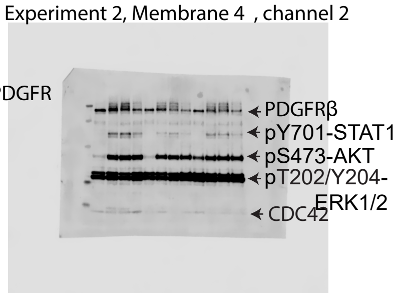

Experiment 2, Membrane 6 , channel 1

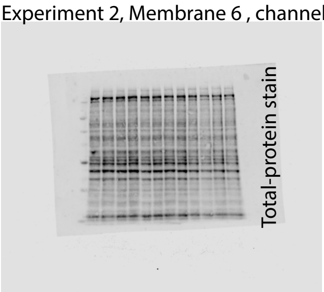

CDC42 knock-down, experiment 3

Experiment 3, Membrane 1, channel 1

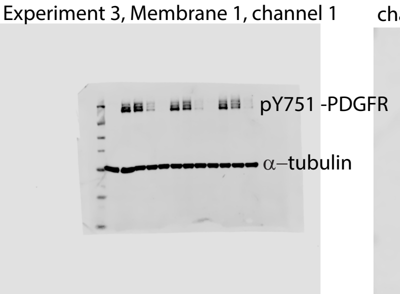

Experiment 3, Membrane 4 , channel 2 (high contrast)

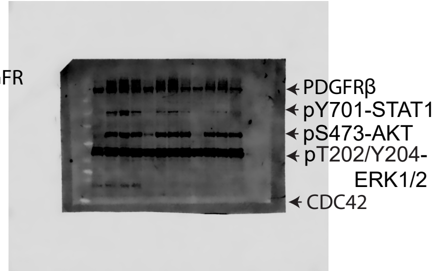

Experiment 3, Membrane 1, channel 2

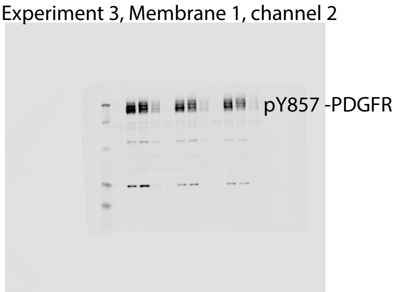

Experiment 3, Membrane 3, channel 1

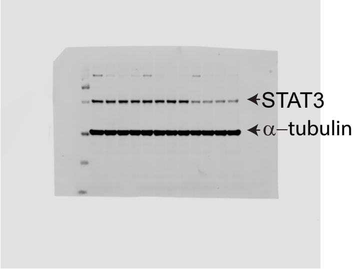

Experiment 3, Membrane 3, channel 2

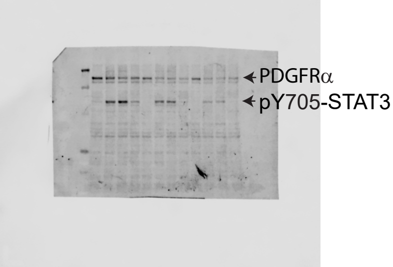

Experiment 3, Membrane 4, channel 1

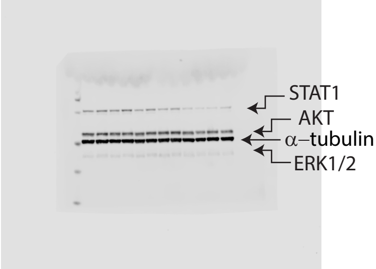

Experiment 3, Membrane 4 , channel 2

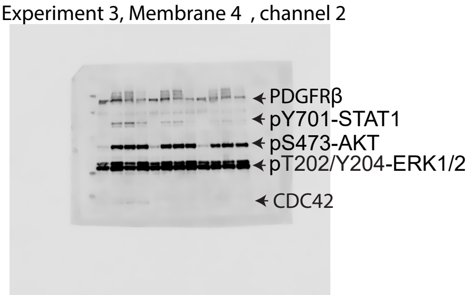

CDC42 knock-down, experiment 4

Experiment , Membrane 1, channel 1

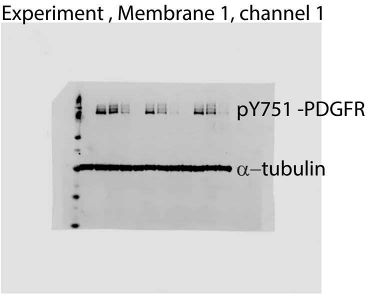

Experiment 4, Membrane 5, channel 2

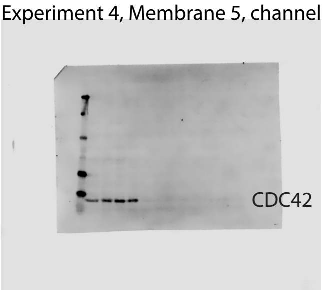

Experiment 4, Membrane 1, channel 2

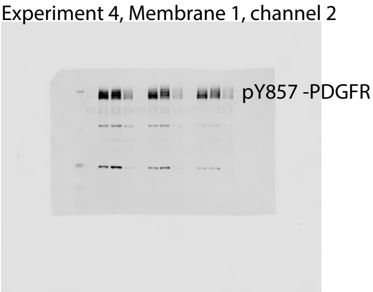

Experiment 4, Membrane 6 , channel 1

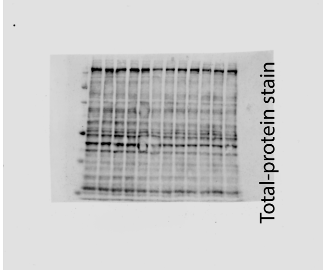

Experiment 4, Membrane 3, channel 1

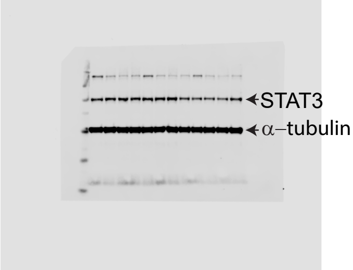

Experiment 4, Membrane 3, channel 2

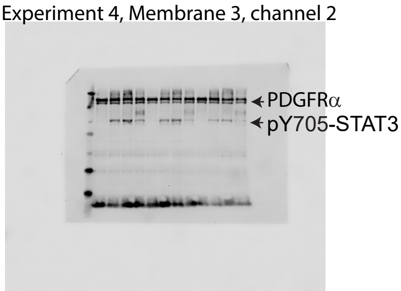

Experiment 4, Membrane 4, channel 1

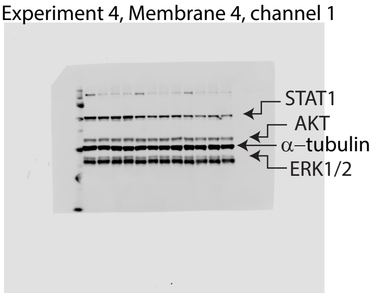

Experiment 4, Membrane 4 , channel 2

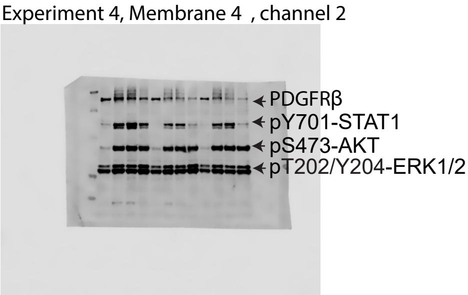

Supplement: Multimedia component 1 [file mmc1.pdf]
