# Supplementary material for: Depletion of the Rho GTPases Cdc42, Rac1 or RhoA reduces PDGF-induced STAT1 and STAT3 signaling
Source: Biochem Biophys Rep. 2024 Sep 25;40:101828. doi: 10.1016/j.bbrep.2024.101828 (PMC11460520; doi:10.1016/j.bbrep.2024.101828)
Supplement: Multimedia component 2 [file mmc2.pdf]

Figure 4 a, full blots

Experiment 1, membrane 1, channel 2

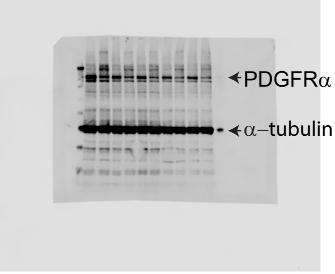

Experiment 2, membrane 1, channel 2

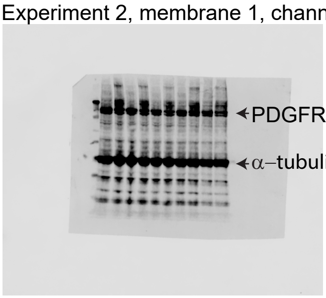

Experiment 3, membrane 1, channel 2

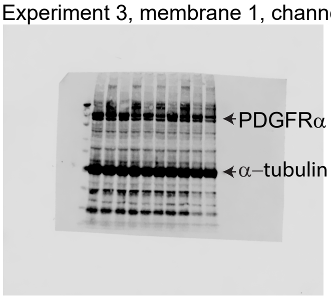

Experiment 4, membrane 1, channel 2

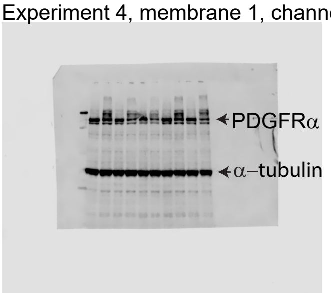

Experiment 1, membrane 2, channel 2

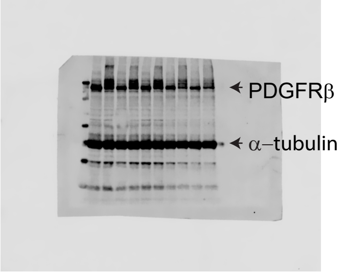

Experiment 2, membrane 2, channel 2

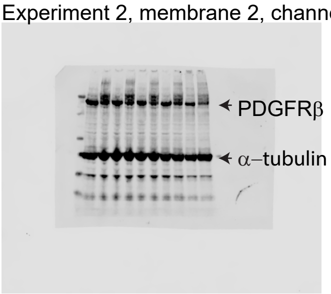

Experiment 3, membrane 2, channel 2

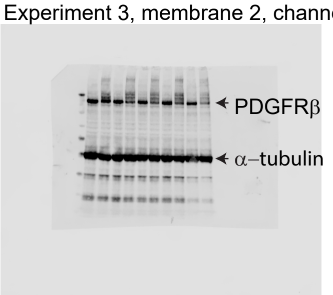

Experiment 4, membrane 2, channel 2

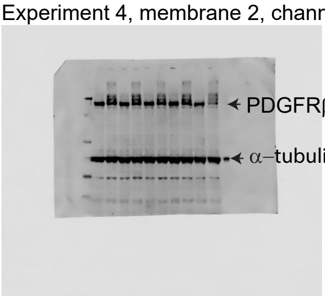

Experiment 1, membrane 3, channel 1

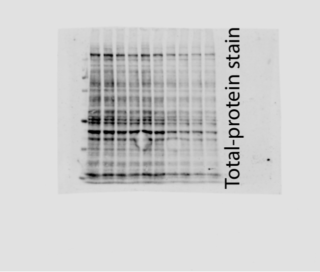

Experiment 2, membrane 3, channel 1

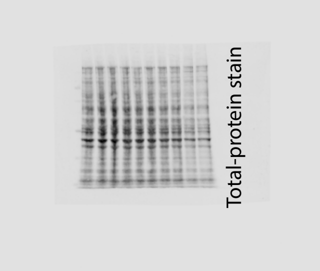

Experiment 3, membrane 3, channel 1

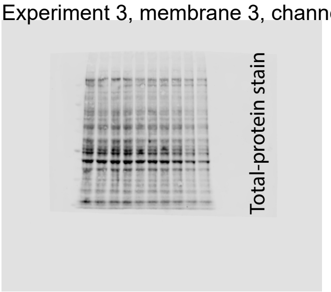

Experiment 4, membrane 3, channel 1

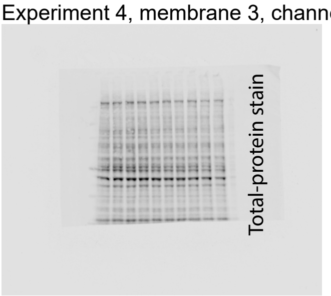

Experiment 5, membrane 1, channel 2

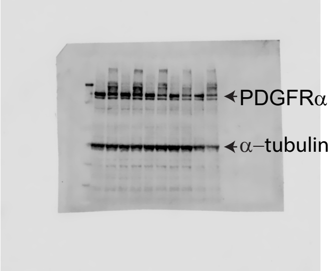

Experiment 5, membrane 2, channel 2

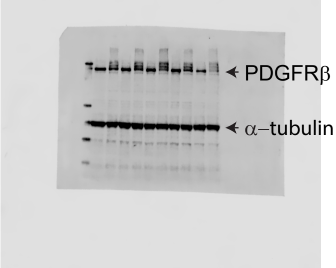

Experiment 5, membrane 3, channel 1

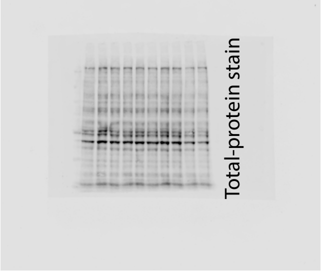

Figure 4 b, full blots

CDC42 knock-down, PDGFRα, experiment 1

Anti-PDGFRα IP

Experiment 1, membrane 1, channel 1

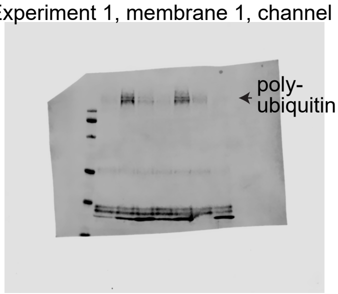

Experiment 1, membrane 1, channel 2

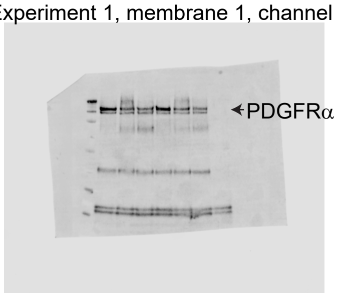

Total cell lysate

Experiment 1, membrane 2, channel 2

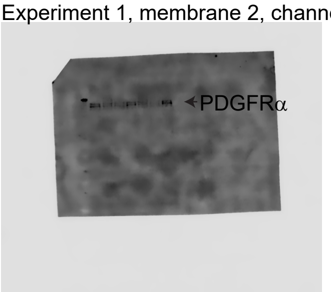

Experiment 1, membrane 3, channel 1

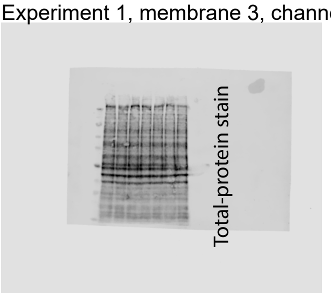

CDC42 knock-down, PDGFRα, experiment 2

Anti-PDGFRα IP

Experiment 2, membrane 1, channel 1

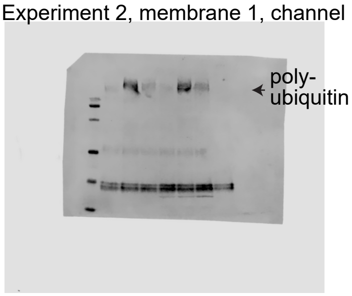

Experiment 2, membrane 1, channel 2

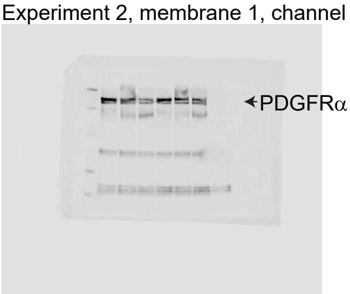

Total cell lysate

Experiment 2, membrane 2, channel 2

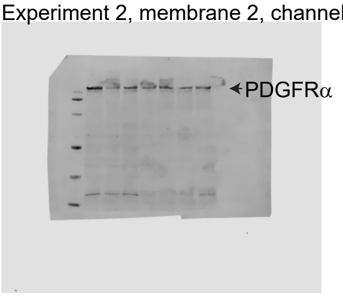

Experiment 2, membrane 3, channel 1

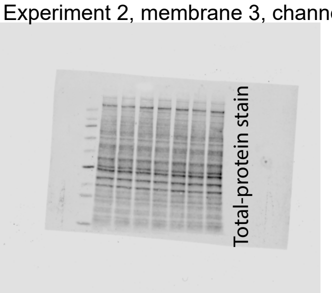

CDC42 knock-down, PDGFRα, experiment 3

Anti-PDGFRα IP

Experiment 3, membrane 1, channel 1

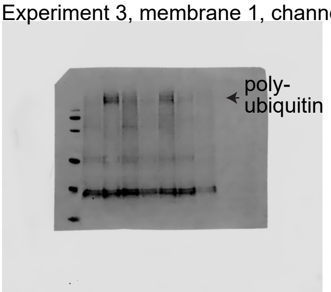

Experiment 3, membrane 1, channel 2

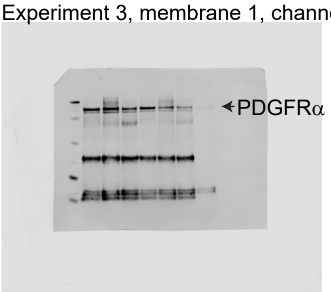

Total cell lysate

Experiment 3, membrane 2, channel 2

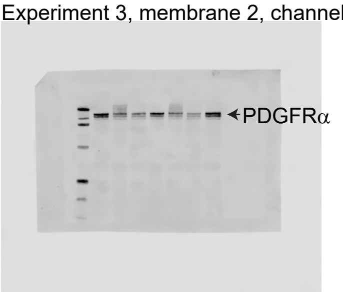

Experiment 3, membrane 3, channel 1

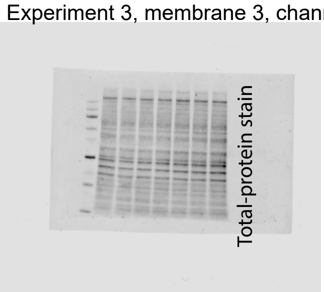

CDC42 knock-down, PDGFRα, experiment 4

Anti-PDGFRα IP

Experiment 4, membrane 1, channel 1

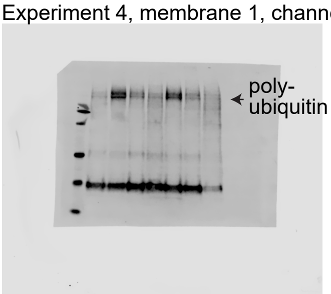

Experiment 4, membrane 1, channel 2

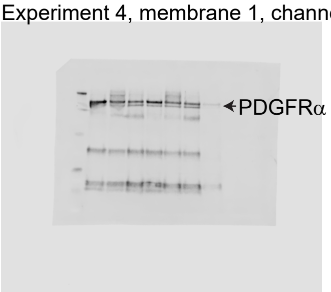

Total cell lysate

Experiment 4, membrane 2, channel 2

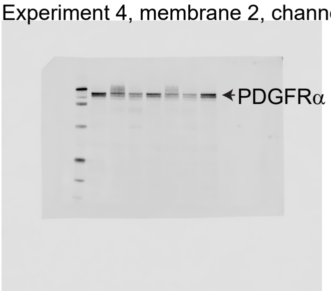

Experiment 4, membrane 3, channel 1

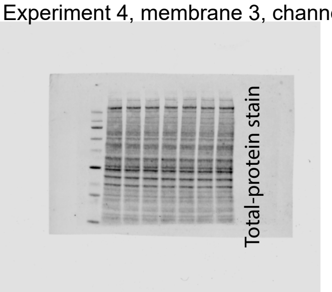

CDC42 knock-down, PDGFRβ, experiment 1

Anti-PDGFRβ IP

Experiment 1, membrane 1, channel 1

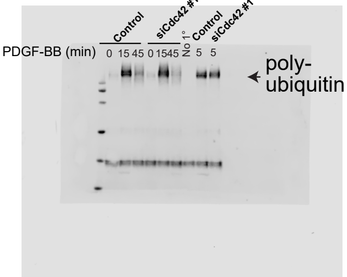

Experiment 1, membrane 1, channel 2

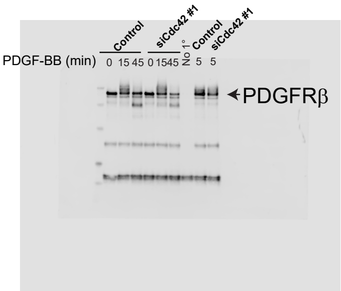

Total cell lysate

Experiment 1, membrane 2, channel 2

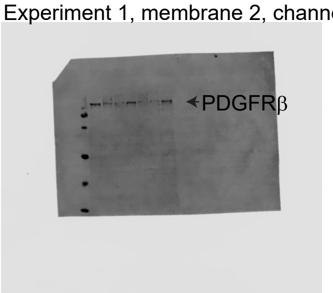

CDC42 knock-down, PDGFRβ, experiment 2

Anti-PDGFRβ IP

Experiment 2, membrane 1, channel 1

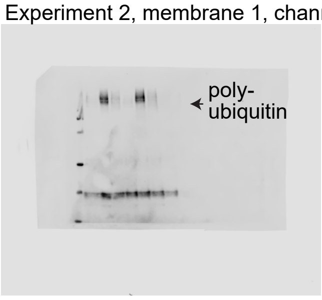

Experiment 2, membrane 1, channel 2

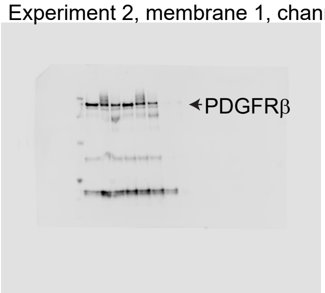

Total cell lysate

Experiment 2, membrane 2, channel 2

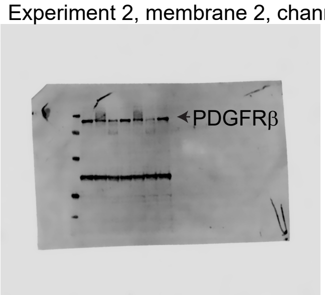

Experiment 2, membrane 3, channel 1

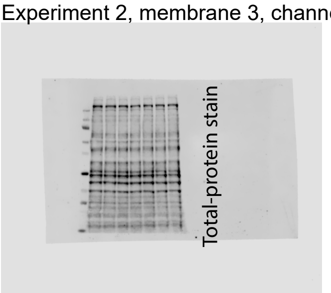

CDC42 knock-down, PDGFRβ, experiment 3

Anti-PDGFRβ IP

Experiment 3, membrane 1, channel 1

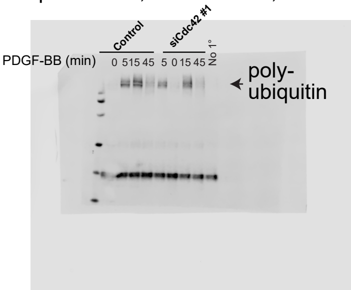

Experiment 3, membrane 1, channel 2

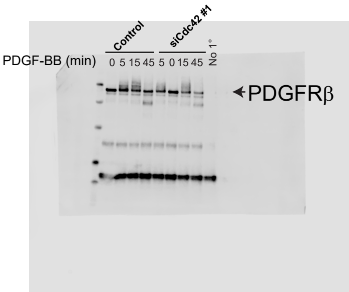

Total cell lysate

Experiment 3, membrane 2, channel 2

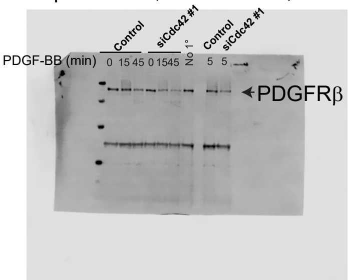

Figure 4 c, full blots

CDC42 knock-down, experiment 1

Biotin-streptavidin PD

Experiment 1, membrane 1, channel 1

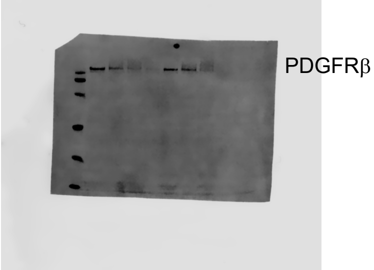

Experiment 1, membrane 1, channel 1

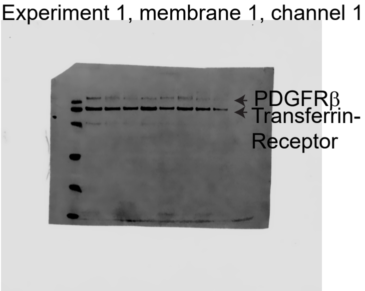

Experiment 1, membrane 1, channel 2

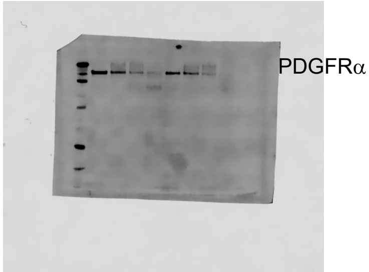

Total cell lysate

Experiment 1, membrane 2, channel 1

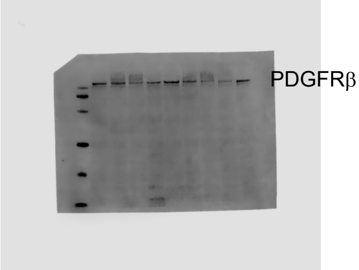

Experiment 1, membrane 2, channel 1

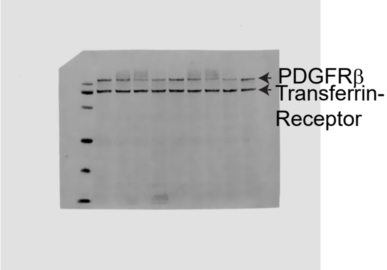

Experiment 1, membrane 2, channel 2

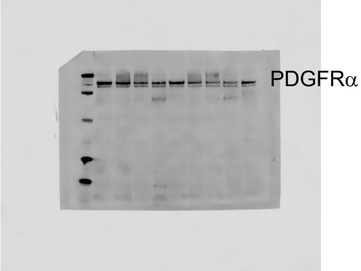

Experiment 1, membrane 3, channel 1

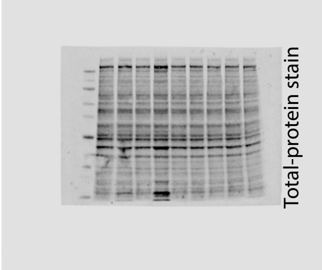

Rac1 and RhoA knock-down, experiment 1

Biotin-streptavidin PD

Experiment 1, membrane 4, channel 1

PDGFRβ

Experiment 1, membrane 4, channel 1

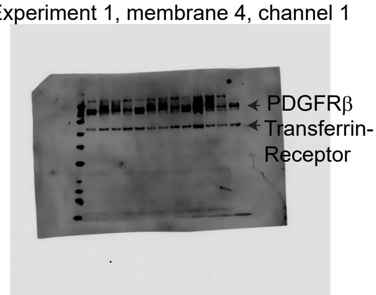

Experiment 1, membrane 1, channel 2

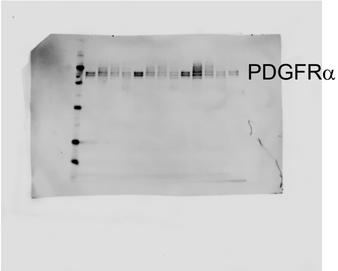

Total cell lysate

Experiment 1, membrane 5, channel 1

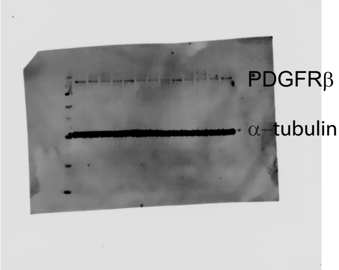

Experiment 1, membrane 5, channel 1

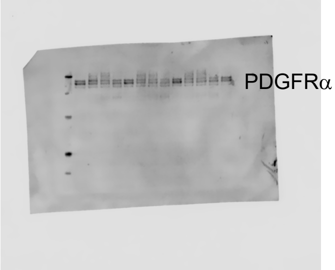

Experiment 1, membrane 6, channel 2

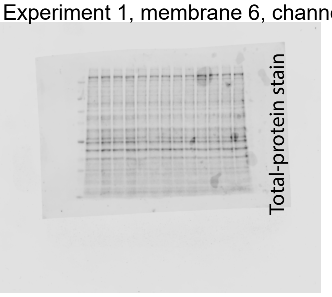

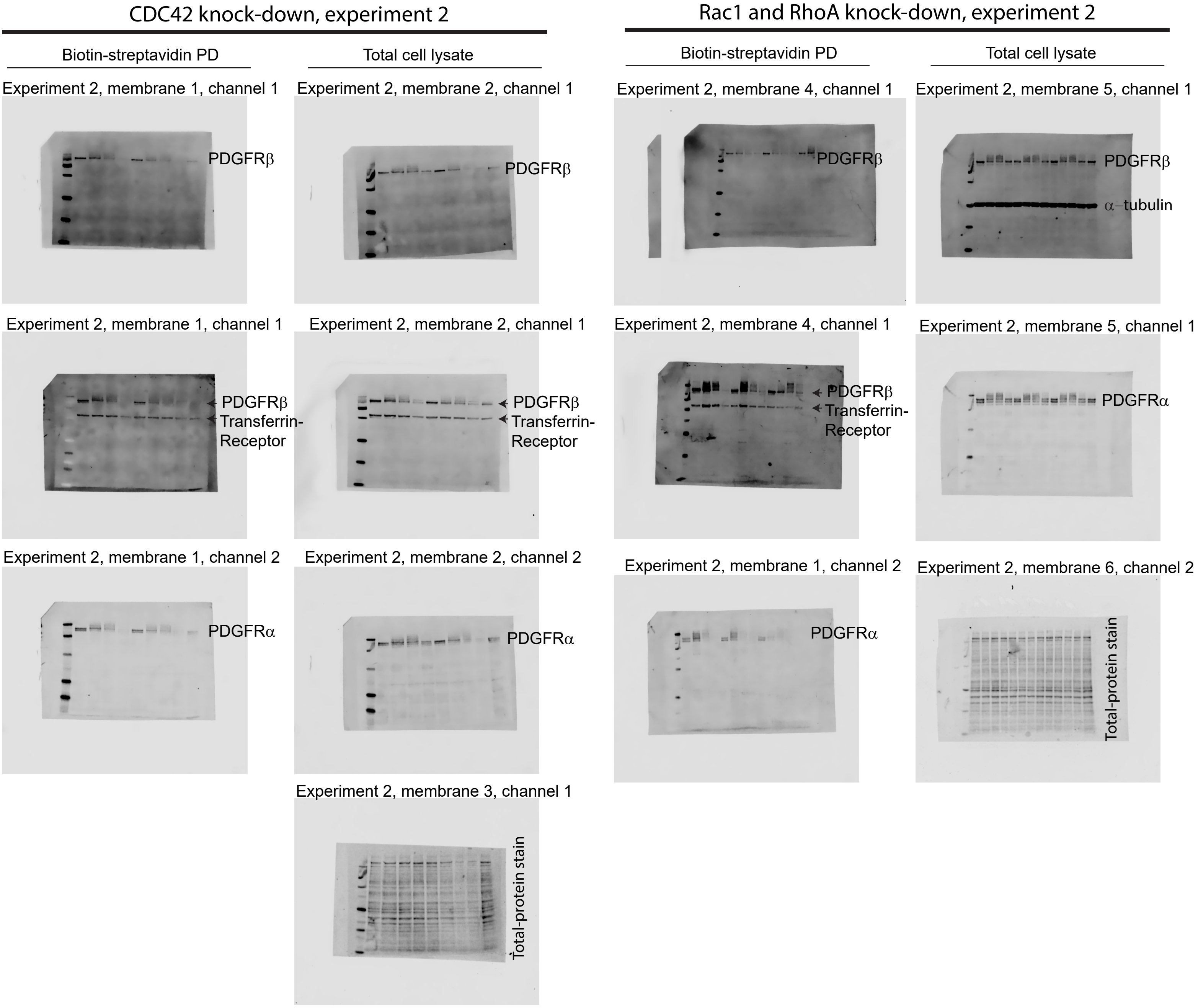
CDC42 knock-down, experiment 2
Biotin-streptavidin PD
Total cell lysate
Experiment 2, membrane 1, channel 1
PDGFRβ
Experiment 2, membrane 2, channel 1
PDGFRβ
Experiment 2, membrane 1, channel 1
PDGFRβ
Transferrin-Receptor
Experiment 2, membrane 2, channel 1
PDGFRβ
Transferrin-Receptor
Experiment 2, membrane 1, channel 2
PDGFRα
Experiment 2, membrane 2, channel 2
PDGFRα
Experiment 2, membrane 3, channel 1
Total-protein stain
Rac1 and RhoA knock-down, experiment 2
Biotin-streptavidin PD
Total cell lysate
Experiment 2, membrane 4, channel 1
PDGFRβ
Experiment 2, membrane 5, channel 1
PDGFRβ
α–tubulin
Experiment 2, membrane 4, channel 1
PDGFRβ
Transferrin-Receptor
Experiment 2, membrane 5, channel 1
PDGFRα
Experiment 2, membrane 1, channel 2
PDGFRα
Experiment 2, membrane 6, channel 2
Total-protein stain

CDC42 knock-down, experiment 3

Biotin-streptavidin PD

Experiment 3, membrane 1, channel 1

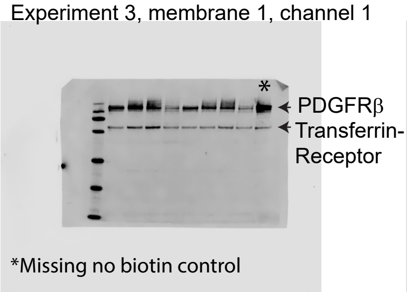

Experiment 3, membrane 1, channel 2

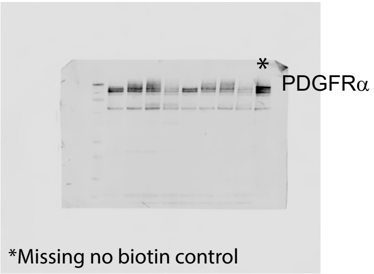

Total cell lysate

Experiment 3, membrane 2, channel 1

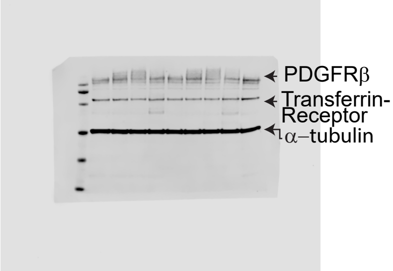

Experiment 3, membrane 2, channel 2

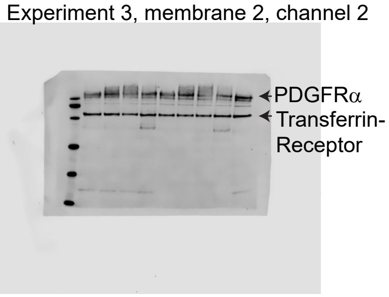

Experiment 2, membrane 3, channel 1

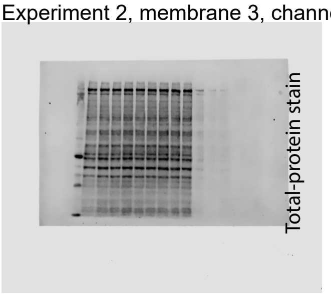

Rac1 and RhoA knock-down, experiment 3

Biotin-streptavidin PD

Experiment 3, membrane 4, channel 1

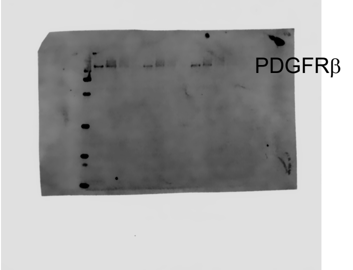

Experiment 3, membrane 4, channel 1

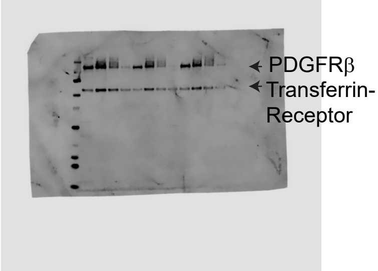

Experiment 3, membrane 1, channel 2

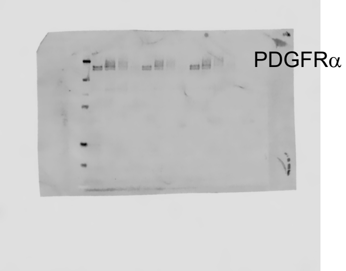

Total cell lysate

Experiment 3, membrane 5, channel 1

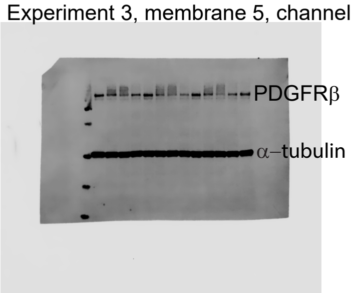

Experiment 3, membrane 5, channel 1

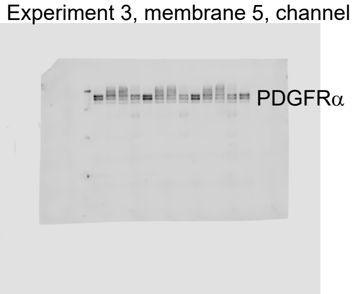

Experiment 3, membrane 6, channel 2

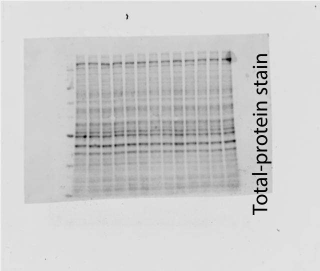

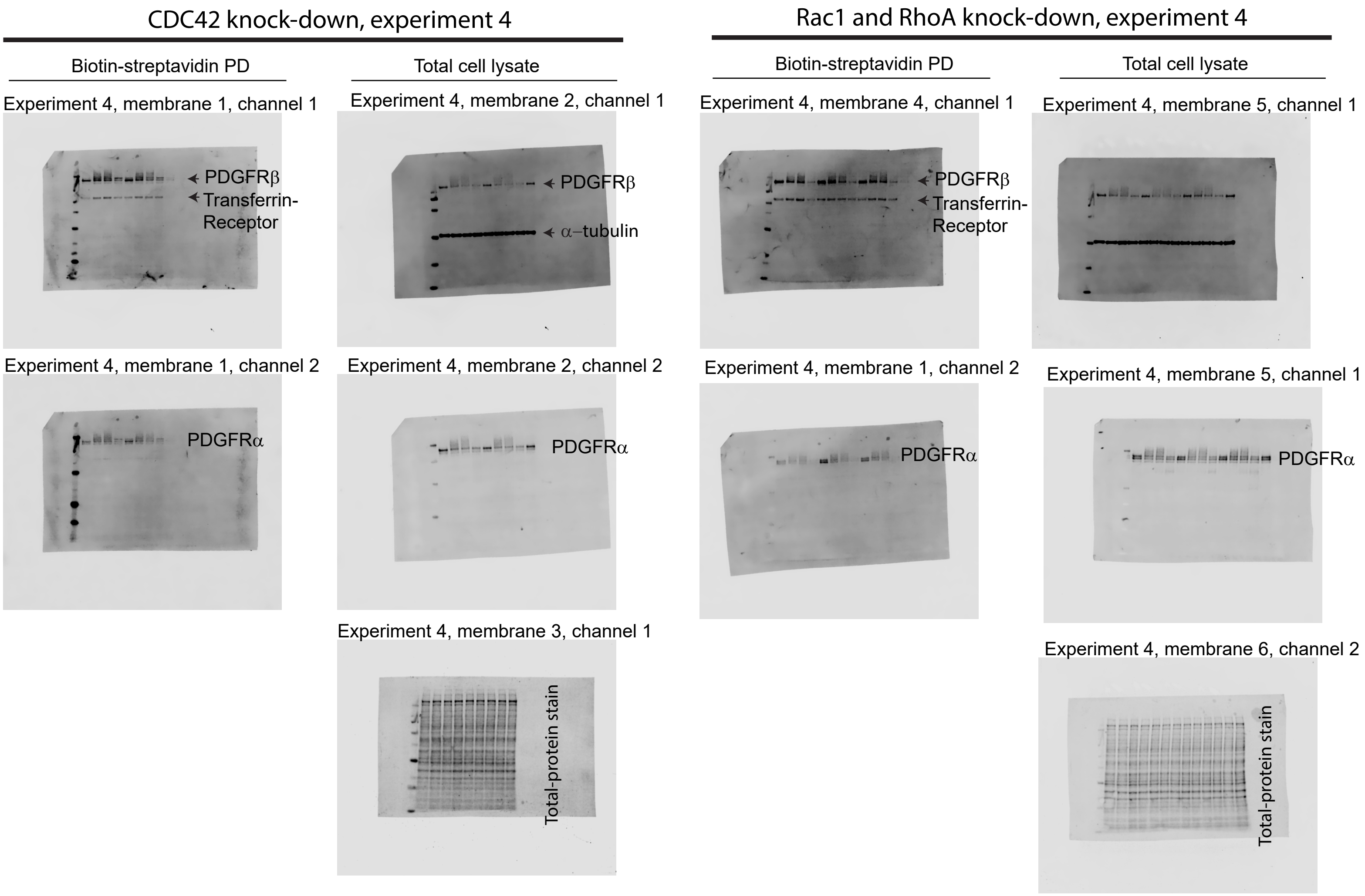
CDC42 knock-down, experiment 4
Biotin-streptavidin PD
Total cell lysate
Experiment 4, membrane 1, channel 1
PDGFRβ
Transferrin-Receptor
Experiment 4, membrane 1, channel 2
PDGFRα
Experiment 4, membrane 2, channel 1
PDGFRβ
α–tubulin
Experiment 4, membrane 2, channel 2
PDGFRα
Experiment 4, membrane 3, channel 1
Total-protein stain
Rac1 and RhoA knock-down, experiment 4
Biotin-streptavidin PD
Total cell lysate
Experiment 4, membrane 4, channel 1
PDGFRβ
Transferrin-Receptor
Experiment 4, membrane 1, channel 2
PDGFRα
Experiment 4, membrane 5, channel 1
Experiment 4, membrane 5, channel 1
PDGFRα
Experiment 4, membrane 6, channel 2
Total-protein stain
